# Treatment with Minicircle DNA Expressing a FGF23 Fragment in a Clinically relevant Mouse Model of X‐Linked Hypophosphatemic Rickets

**DOI:** 10.1002/advs.202508870

**Published:** 2025-10-31

**Authors:** Huixiao Wu, Wanyi Zhao, Xinyu Chen, Yanzhou Wang, Shuoshuo Wei, Bo Xiang, Yingzhou Shi, Zongyue Li, Yangyang Yao, Jin Xie, Renyuan Qiu, Meng Shu, Shuo Xu, Ping Chen, Zhiying Chen, Yingjie Wu, Weibo Xia, Ling Gao, Yongfeng Song, Jiajun Zhao, Chao Xu

**Affiliations:** ^1^ Key Laboratory of Endocrine Glucose & Lipids Metabolism and Brain Aging, Ministry of Education; Department of Endocrinology and Metabolism Shandong Provincial Hospital Affiliated to Shandong First Medical University Jinan 250021 P. R. China; ^2^ Shandong Provincial Research Center for Stem Cells and Gene Therapy of Endocrine Metabolic Diseases Jinan 250021 P. R. China; ^3^ Institute of Endocrinology Shandong Academy of Clinical Medicine Jinan 250021 P. R. China; ^4^ Shandong Clinical Medical Center of Endocrinology and Metabolism Jinan 250021 P. R. China; ^5^ Department of Pediatric Orthopedics Shandong Provincial Hospital Affiliated to Shandong First Medical University Jinan 250021 P. R. China; ^6^ Department of Radiology Shandong Rongjun General Hospital Jinan 250013 P. R. China; ^7^ Department of Medical imaging Shandong Provincial Hospital Affiliated to Shandong First Medical University Jinan 250021 P. R. China; ^8^ Syno Minicircle Biotechnology Co. Ltd. Shenzhen 518107 P. R. China; ^9^ College of Laboratory Animals (Shandong Laboratory Animal Center) Medical Science and Technology Innovation Center Shandong Provincial Hospital Shandong First Medical University & Shandong Academy of Medical Sciences Jinan 250117 P. R. China; ^10^ Department of Endocrinology Peking Union Medical College Hospital Chinese Academy of Medical Sciences & Peking Union Medical College Beijing 100730 P. R. China

**Keywords:** gene therapy, minicircle DNA, mouse model, PHEX, XLHR

## Abstract

X‐linked hypophosphatemic rickets (XLHR) is a rare X‐linked dominant skeletal dysplasia caused by phosphate regulating endopeptidase homolog X‐linked (PHEX) gene mutation. Until now, the pathogenic role of PHEX has not been fully determined, and there has been no radical cure for XLHR. In the previous study, a novel *PHEX* variant (c.T1349C; p.L450P) is identified in a child with XLHR. The present study aims to reveal its pathogenic role and evaluate the therapeutic effects of the minicircle (MC)‐DNA in XLHR. In vitro, the wildtype and mutant plasmids are introduced into HEK293 cells. In vivo, a new knock‐in XLHR mouse model carrying the novel variant is established. Furthermore, this study makes the first attempt to perform gene therapy using a MC‐DNA vector expressing a fragment of FGF23 (amino acids 180‐251) in the *Phex*‐T1349C mice. The new mouse model demonstrates the clinical manifestations of XLHR seen in the patient, including a gene dosage effect. Furthermore, MC‐DNA is found to slightly increase blood phosphorus levels, significantly decrease serum alkaline phosphatase levels, and improve bone mineralization without apparent adverse effects for at least 6 weeks. This study suggests MC‐DNA as a promisingly safe and effective therapeutic strategy to treat XLHR.

## Graphical abstract

The pathogenic role of PHEX isn't fully determined, and there is no radical cure for X‐linked hypophosphatemic rickets (XLHR). This study makes the first attempt to perform gene therapy using a minicircle DNA (MC‐DNA) vector expressing a fragment of FGF23 (amino acids 180‐251) in *Phex*‐T1349C mice and suggests MC‐DNA as a promisingly safe and effective therapeutic strategy to treat XLHR.

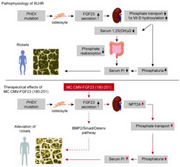

## 1 Introduction

X‐linked hypophosphatemic rickets (XLHR) has been identified as the most prevalent form of inherited rickets. XLHR is a dominantly inherited disease caused by mutations of the *PHEX* gene, with a prevalence of 1 in 20000 individuals.^[^ ^1^ ^]^ It is characterized by hypophosphatemia, aberrant vitamin D metabolism, growth retardation and bone mineralization defects, and is associated with a pathological elevation of fibroblast growth factor 23 (FGF23). The phosphate‐regulating gene with homologies to endopeptidases on the X chromosome (*PHEX*) gene comprises 22 exons and encodes a transmembrane endopeptidase belonging to the type II integral membrane zinc‐dependent endopeptidase family. PHEX is composed of 749 amino acids and contains three domains: a short cytoplasmic N‐terminal region (amino acids 1‐20), a single transmembrane domain (amino acids 21‐37), and a long extra‐cytoplasmic domain (amino acids 38‐749), which contains zinc‐binding motifs as the active site of the enzyme.^[^ ^1^ ^]^ To date, the Human Gene Mutation Database (HGMD) has reported more than 640 mutations of *PHEX* gene that are associated with XLHR. However, only a limited number of these mutations have been evaluated for their pathogenic role and only by in vitro studies.^[^ ^2^, ^3^, ^4^ ^]^

Patients with XLHR present with elevated FGF23 levels, which is currently considered to be an important phosphorus‐regulating factor involved in a bone–kidney axis regulating phosphate homeostasis and matrix mineralization.^[^ ^5^, ^6^, ^7^, ^8^ ^]^ However, the role of FGF23 in the pathophysiology of XLHR is incompletely understood. Furthermore, the precise mechanisms through which mutations in *PHEX* lead to alterations in FGF23 levels remain to be fully elucidated. XLHR is frequently characterized by an early onset, with overt clinical symptoms and a high incidence of disability. Current clinical management of XLHR frequently relies on conventional Pi supplementation in conjunction with active vitamin D analogs, a practice that has been observed to demonstrate limited efficacy and the potential to induce adverse effects, including an elevated risk of hyperparathyroidism, nephrocalcinosis, and concurrent increases in circulating FGF23 levels, which may potentially mitigate the therapeutic benefits of this approach.^[^ ^9^, ^10^ ^]^ Recently, a novel therapy for XLHR has been approved: the humanised monoclonal FGF23 antibody burosumab (trade name: CRYSVITA), which can directly counteract the effects of FGF23. Burosumab has been employed in the treatment of both pediatric and adult patients with XLHR, with studies demonstrating its efficacy in restoring phosphorus homeostasis and ameliorating the severity of rickets.^[^ ^11^, ^12^, ^13^, ^14^, ^15^, ^16^ ^]^ However, its widespread application is limited by cost considerations, and its long‐term efficacy and safety profile remains to be fully established. Consequently, the development of new therapeutic approaches that are both effective and safe for the treatment of XLHR remains a clinical necessity.

As the majority of inherited human diseases are monogenic, gene therapy involving the correction of mutated genes or site‐specific modifications is considered to be a potential permanent therapeutic strategy.^[^ ^17^, ^18^ ^]^ A plethora of vectors are utilized for the delivery of target genes during such therapeutic interventions. Among these, adeno‐associated viruses (AAV) predominate due to their high transduction efficiency and sustained expression levels of their cargo.^[^ ^19^, ^20^ ^]^ Recently, it has been shown that a liver‐targeting AAV vector expressing the C‐terminal tail of FGF23 corrected skeletal manifestations and osteomalacia in a XLHR mouse model.^[^ ^21^ ^]^ Nevertheless, an immune response that counteracts the efficacy of such approaches remains the biggest unresolved issue in their implementation.^[^ ^22^, ^23^ ^]^ Moreover, treatment toxicity, limited cloning capacity of viral vectors, and their high production costs are hurdles that also prevent their widespread use. In contrast, nonviral vectors offer distinct advantages, including their ability to accommodate unlimited packaging capacity, cost‐effectiveness, and reduced immunogenicity.^[^ ^24^, ^25^ ^]^ But the low level and the short duration of gene expression offered by such nonviral vectors also limits their clinical application. Recent studies have indicated that minicircle (MC)‐DNA vectors, representing a novel form of supercoiled DNA containing a minimal expression cassette resulting from a state‐of‐the‐art in vivo recombination process to excise prokaryotic sequences, may circumvent biological safety concerns and transgene silencing from plasmid DNA (pDNA).^[^ ^26^, ^27^, ^28^, ^29^, ^30^ ^]^ Furthermore, MC‐DNA vectors have demonstrated both enhanced transgene expression and persistence in comparison with conventional plasmids.^[^ ^31^, ^32^, ^33^, ^34^ ^]^

In this study, we sought to ascertain the pathogenic role of a novel *PHEX* variant (c.T1349C; p.L450P) through a combination of in vitro and in vivo experiments. We successfully established a novel mouse line (Phex‐T1349C),^[^ ^35^ ^]^ which carries a missense mutation in exon 12 of the *Phex* gene resulting from a substitution of leucine with proline, akin to that observed in the patient at amino acid 450. The efficacy of a novel approach involving the administration of a MC‐DNA vector expressing a fragment of FGF23 was investigated in this novel model. The results demonstrated a significant decrease in serum alkaline phosphatase levels and an improvement in bone mineralization, with no apparent adverse effects observed for a period of at least 6 weeks. These findings underscore the significant contribution of this novel model to the existing mouse models of XLHR, offering novel pathogenic insights into the role of PHEX in bone mineralization. Furthermore, these results suggest MC‐DNA as a potential efficacious and safe treatment for XLHR.

## 2 Results

### 2.1 Identification of a Novel *PHEX* Missense Variant in a Patient with XLHR

The patient, a 14‐year‐old Chinese boy, was initially admitted to our hospital on the grounds of genu varum and waddling gait. He was only 136 cm (−3.04 SD) tall at the first clinical evaluation. Initial clinical assessments revealed biochemical and hormonal imbalances, characterized by hypophosphatemia, hyperparathyroidism, vitamin D deficiency, hyperalkaline phosphatasemia, and elevated levels of FGF23 and its co‐receptor α‐Klotho, while serum calcium levels remained within normal parameters (Table S1, Supporting Information). Radiographs showed genu varum and metaphyseal widening and irregularity (Figure S1, Supporting Information). It is noteworthy that the patient's mother also exhibited short stature and genu varum.

Subsequently, whole‐exome sequencing analysis of the patient was conducted, and gene variants were confirmed via Sanger sequencing in all available family members. The patient was found to carry a novel heterozygous mutation c.1349T>C (p.L450P) in *PHEX*, which was inherited from his mother (Figure S2A, Supporting Information). This variant has not been reported previously in the HGMD, TOPMED, ExAC, and 1000Genome databases, suggesting that the mutation identified in this patient is both novel and rare.

The novel c.1349T>C (p.L450P) variant was located in the extracellular domain, with leucine being replaced by proline at amino acid 450 without altering the length of the protein (Figure S2B, Supporting Information). This single base alteration was strongly predicted to be pathogenic and deleterious using two online bioinformatic software packages—MutationTaster and PROVEAN—and it affected a highly conserved amino acid residue in diverse species by sequence alignment (Figure S2C, Supporting Information), strongly suggesting that the mutation has a disease‐causing effect. Subsequently, the protein model of mutant and wild‐type PHEX was conducted by I‐TASSER and visualized by the PyMOL viewer. The mutation site p.L450P was shown to be relatively poorly packed and some secondary structure sites were also altered in the extracellular region (Figure S2D, Supporting Information). These alterations may affect the posttranslational modification of the PHEX protein or its interaction with other proteins. The data presented herein suggests that the novel mutation is a pathogenic variant for XLHR.

### 2.2 T1349C Variant is a Loss‐of‐Function Variant In Vitro

**Figure 1:**
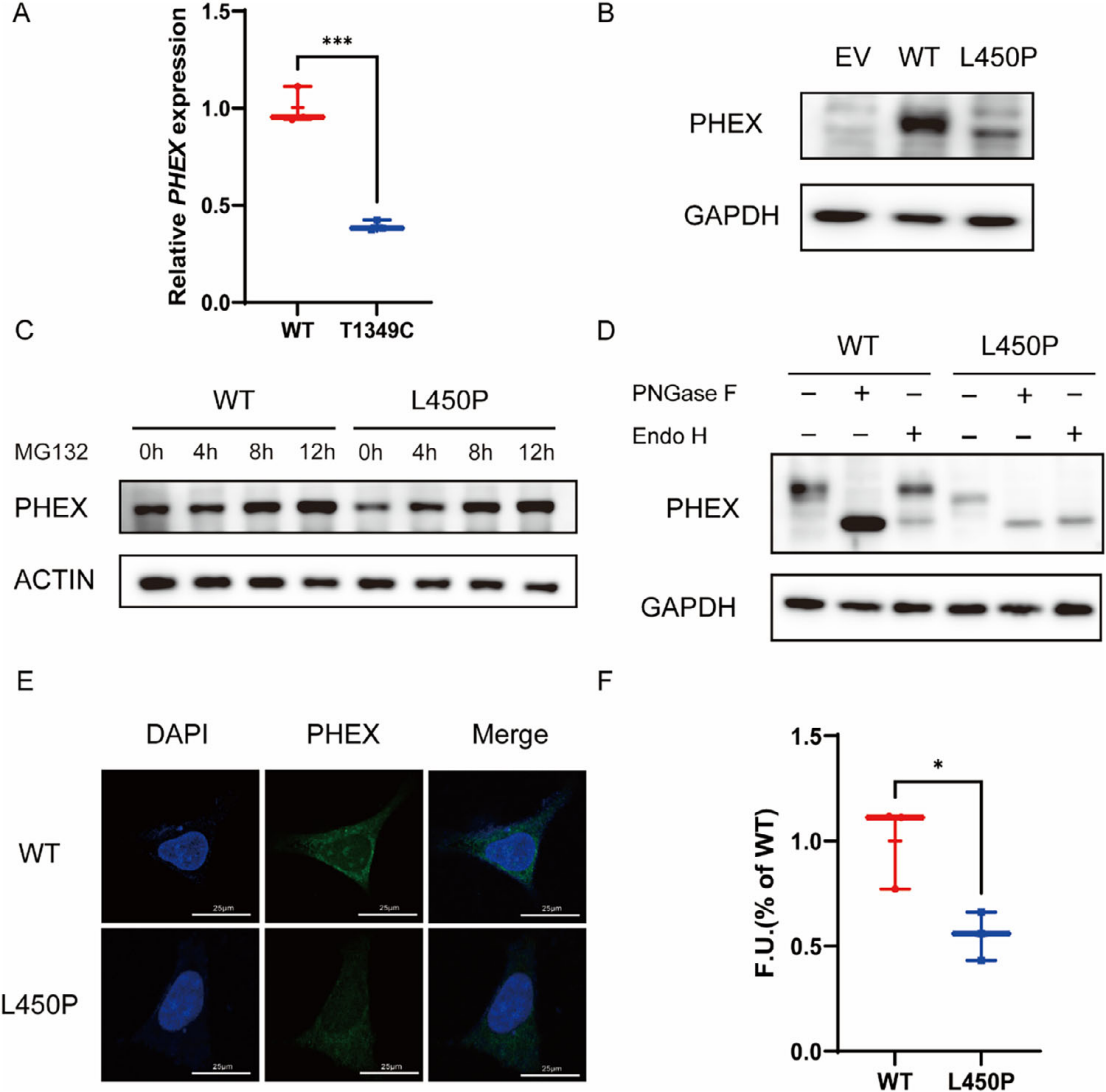
Functional analysis of the novel *PHEX* variant (c.1349T>C) in vitro. A) Gene expression analysis of wild‐type (WT) and mutant *PHEX* in transiently transfected HEK293 cells by real‐time quantitative polymerase chain reaction (PCR). B) Representative western blot of PHEX from whole‐cell lysates of transiently transfected HEK293 with empty vector (EV), or WT‐ or mutant PHEX‐expressing plasmids. GAPDH antibody was used as a loading control. C) Representative western blot of PHEX expressed in transiently transfected HEK293 cells at baseline and after 4, 8, and 12 h of MG132 treatment. β‐actin antibody was used as a loading control. D) Representative western blot of WT and mutant PHEX from whole‐cell lysates of transiently transfected HEK293 cells incubated with either PNGase F or endo H. The upper band represents the fully glycosylated mature form while the lower band is a core glycosylated or a deglycosylated form. GAPDH antibody was used as a loading control. E) Subcellular localization analysis of WT and PHEX mutants (green) in HEK293 cells 48 h after transfection with expression plasmids. Nuclei were visualized by DAPI. Scale bars, 20 µm. F) The velocity (V, F.U. min^−1^) of endopeptidase activity of WT and mutant secPHEX proteins (200 ng) assessed with a fluorogenic peptide substrate (30 × 10^−6^ m) after incubation at 37 °C for 1 h, as calculated from the linear portion of the curve. Bars depict the mean ± SEM of three independent assays for each *PHEX* variant (*N* = 3), ^*^ *p* < 0.05, ^**^ *p* < 0.001, ^***^ *p* < 0.0001 by two‐tailed Student's *t*‐test.

To further confirm the pathogenicity of this novel missense variant, we investigated its effect on PHEX protein expression, protein maturation, cellular trafficking, and endopeptidase activity. First, we tested the overall expression levels of WT and mutant PHEX in HEK293 cells transfected with vectors expressing FLAG‐WT‐PHEX or FLAG‐MUT‐PHEX. We found that both the mRNA (**Figure** **1**A) and protein expression (Figure 1B) of mutant PHEX were significantly lower than that of the WT counterparts, which may be attributed to the impeded production or increased degradation of mutant PHEX. We next investigated the stability of wild‐type PHEX and mutant PHEX proteins by using a MG132 (Z‐Leu‐Leu‐Leu‐al) protein degradation assay. We transfected HEK‐293 cells with FLAG‐WT‐PHEX or FLAG‐MUT‐PHEX vectors and then incubated the cells at four different time points (baseline, 4, 8, and 12 h) with 1 × 10^−3^ m of MG132, a known inhibitor of the 20S proteasome. We found that wild‐type PHEX remained relatively stable even after 12 h, whereas the mutant PHEX protein showed obvious increase after 8 h, indicating PHEX^T1349C^ is more susceptible to proteasome degradation (Figure 1C). We then carried out a deglycosylation experiment to analyze protein maturation using two different glycosylases, PNGase F and Endo H. This treatment allows for the discrimination between core glycosylated proteins present in the endoplasmic reticulum (ER) and fully glycosylated proteins that have passed through the compartments of the Golgi apparatus. Both wild‐type PHEX and the L450P mutant were completely sensitive to PNGase F digestion and a single protein band of 87 kDa was apparent after removal of N‐linked oligosaccharide side chains (Figure 1D). However, when treated with Endo H, most of the wild‐type protein was resistant to Endo H digestion, whereas the L450P mutant was susceptible. In addition, we tested the subcellular localization of each form of PHEX by immunofluorescence assay, and we found that both WT and the L450P mutant could be detected in permeabilized cells but the expression of mutant PHEX was markedly lower (Figure 1E), consistent with the western blot result. However, the cellular localization of PHEX was unchanged, indicating that the novel variant did not affect the localization of PHEX to the cell membrane (Figure 1E). Finally, we transfected HEK293 cells with wild‐type or mutant secPHEX plasmids and collected secreted PHEX in the cell media for protein quantification. A protein band of approximately 95 kDa was detected in the media of cells expressing WT and L450P secPHEX plasmids. As a member of the M13 subfamily, PHEX is similar to neutral endopeptidase (NEP), so we tested the catalytic activity of secPHEX and found that the L450P mutant exhibited a significantly lower level of activity (approximately 45% of wild‐type secPHEX activity) (Figure 1F). Taken together, these results confirm that the c.1349T>C *PHEX* mutation is a loss‐of‐function allele.

### 2.3 *Phex*‐T1349C Mice Present with Clinically Relevant Phenotypes of XLHR

**Figure 2:**
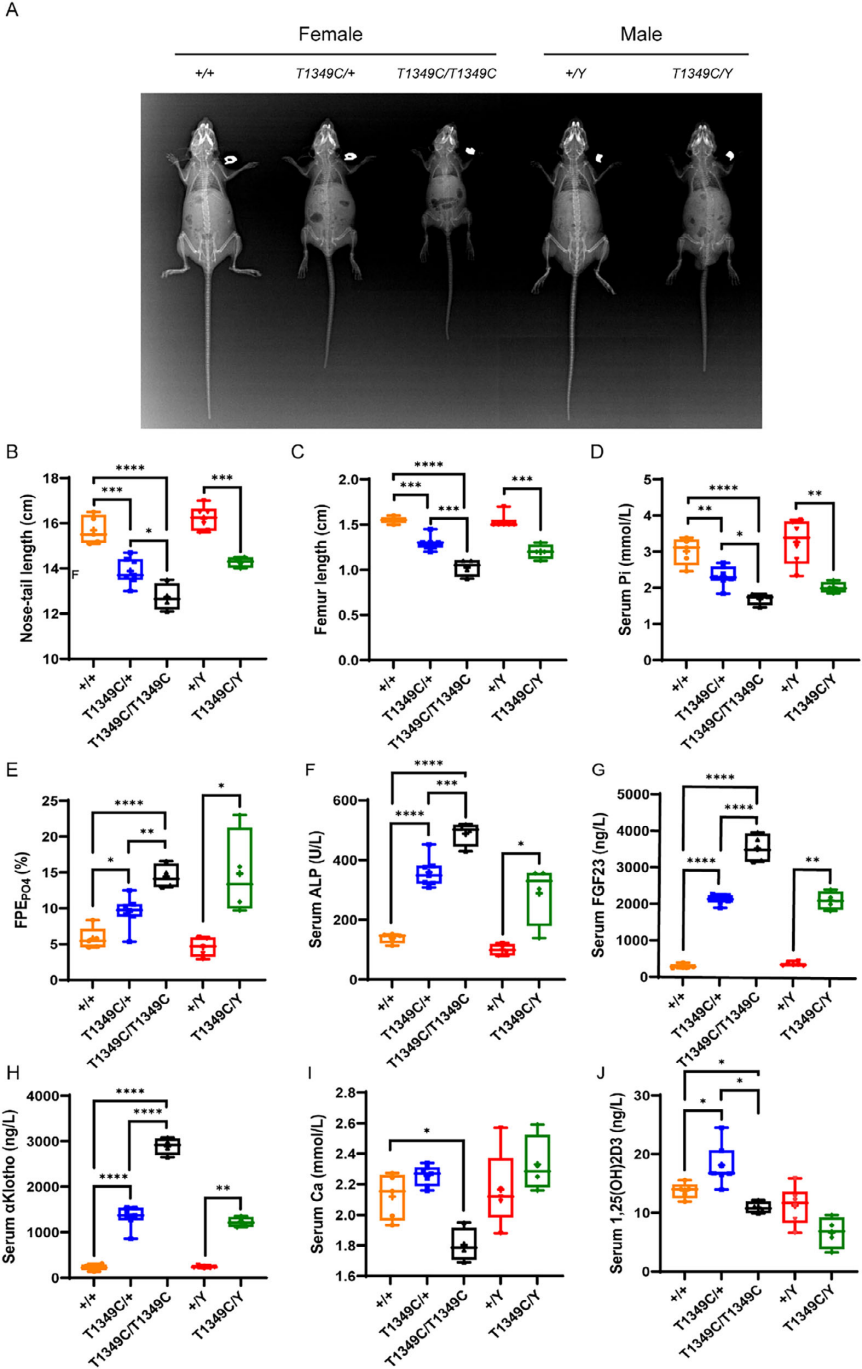
Growth characteristics, serum biochemistry and urine phosphate excretion of *Phex*‐T1349C mice. A) Faxitron X‐ray images of wild‐type (WT) and *Phex*‐T1349C mice at 12 weeks of age. B) Body length of age‐matched (12‐week‐old), sex‐matched mutant and WT mice. C) Femoral length of age‐matched (12‐week‐old), sex‐matched mutant and WT mice. Serum phosphorus (D), urine fractional phosphate excretion (FPE_PO4_) (E), serum alkaline phosphatase (ALP) (F), FGF23 (G), α‐Klotho (H), calcium (I) as well as 1,25‐(OH)_2_D levels (J). Samples were taken at sacrifice from age‐matched (12‐week‐old), sex‐matched *Phex*‐T1349C and WT mice. For box‐and‐whiskers plots, the center line represents the median, the bounds of the box represent quartiles and the whiskers represent min to max (*N* = 3–7 each group), ^*^ *p* < 0.05, ^**^ *p* < 0.01, ^***^ *p* < 0.001, ^****^ *p* < 0.0001 by two‐tailed Student's *t*‐test or one‐way ANOVA for normally distributed continuous variables with homogeneous variance, or Mann Whitney test for non‐normally distributed continuous variables with nonhomogeneous variance.

Having confirmed the pathogenic role of the c.1349T>C variant through in vitro assays, we next generated a new knock‐in XLHR mouse model carrying the c.1349T>C variant using CRISPR/Cas9 technology. Despite the fully penetrant physical abnormalities of the affected mice, their lifespan and fertility remained equivalent to that of WT littermates. Sequencing of the *Phex* gene of the mutant mice identified a thymine to cytosine substitution (Figure S3A, Supporting Information) in the 47th base pair of exon 12, resulting in the substitution of leucine by proline at amino acid 450. In bones from *Phex* ^T1349C^/+ and from *Phex* ^T1349C^/*Phex* ^T1349C^ females, Phex is present at reduced levels in the heterozygous females, but it is at even lower levels in the homozygous mutant females. In bones from *Phex* ^T1349C^/Y males (as *Phex* is located on the X‐chromosome), mutant Phex is not detectable by immunoblot analysis (Figure S3B, Supporting Information). And as expected from the lower expression of the protein, both hemizygous males (*Phex* ^T1349C^/Y) and heterozygous or homozygous females (*Phex* ^T1349C^/+, *Phex* ^T1349C^/*Phex* ^T1349C^) presented with typical XLHR phenotypes from 3 weeks of age (data not shown); namely their gross appearance included a smaller body size and shortened hind limbs and tail (Figure S3C, Movies S1 and S2, Supporting Information). In addition, X‐ray analysis of both male and female mice at 12 weeks of age clearly revealed skeletal abnormalities consistent with rickets, including severe osteopenia of the entire skeleton, shortened long bones, abnormal curvature of the pelvis and long bones, and dental dysplasia (**Figure** **2**A–C). And the variant is fully penetrant, as affected males do not sire unaffected females.

As another hallmark of XLHR is hypophosphatemia, we next analyzed serum parameters related to phosphate metabolism. The results showed that phosphorus (Pi) levels were significantly lower (Figure 2D), but urinary fractional excretion of phosphorus (FE_PO4_) (Figure 2E), serum alkaline phosphatase (ALP) (Figure 2F), FGF23 (Figure 2G), the co‐receptor of FGF23, α‐Klotho (Figure 2H) levels were significantly elevated in mutant hemizygous males and heterozygous/homozygous females compared to age‐ and sex‐matched WT mice. And a clear gene‐dose effect was observed. In contrast to other XLHR mouse models, serum calcium (Ca) levels were not significantly altered in hemizygous males and heterozygous females, but were obviously lower in homozygous females (Figure 2I). In addition, the serum level of 1,25‐(OH)_2_D was significantly higher in heterozygous females and not significantly changed in hemizygous males, while that of homozygous females was significantly lower compared to WT littermates (Figure 2J).

### 2.4 Bone Histomorphometry of *Phex*‐T1349C Mice

**Figure 3:**
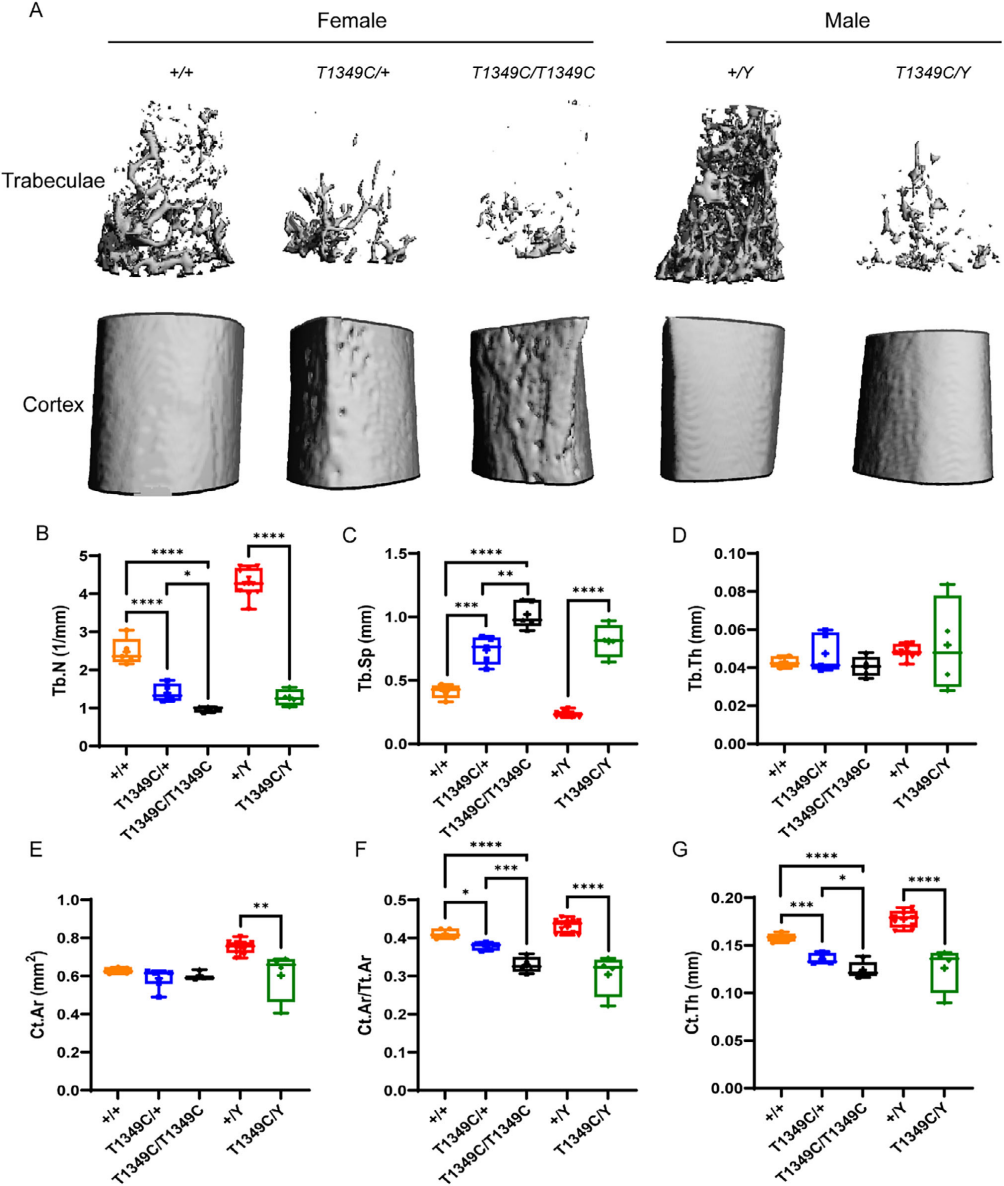
micro‐CT analysis of the femurs of *Phex*‐T1349C mice and wild‐type (WT) littermates. A) The 3D reconstruction images of trabeculae and cortex of the femur. Trabecular bone parameters including trabecular number (Tb.N) (B), trabecular separation (Tb.Sp) (C) and trabecular thickness (Tb.Th) (D) in female and male mice. And cortical bone parameters including cortical bone area (Ct.Ar) (E), cortical bone area/ cortical total area (Ct.Ar/Tt.Ar) (F) and cortical thickness (Ct.Th) (G) in female and male mice. For box‐and‐whiskers plots, the center line represents the median, the bounds of the box represent quartiles and the whiskers represent min to max (*N* = 3–7 each group), ^*^ *p* < 0.05, ^**^ *p* < 0.01, ^***^ *p* < 0.001, ^****^ *p* < 0.0001 by Student's *t*‐test or one‐way ANOVA for normally distributed continuous variables with homogeneous variance, or Mann Whitney test for non‐normally distributed continuous variables with nonhomogeneous variance.

By micro‐CT analysis we found that *Phex*‐T1349C mice had significantly fewer trabeculae and a rough cortical surface compared to WT mice (**Figure** **3**A). At the distal femoral metaphysis, *Phex*‐T1349C mice had significantly fewer trabeculae (Figure 3B) and greater trabecular separation (Figure 3C) in both males and females compared to WT littermates, whereas no difference in trabecular thickness was observed (Figure 3D). At the mid‐diaphysis of the femur, there was no significant change in the cortical bone area (Ct.Ar) in female mice, but the Ct.Ar of hemizygous males was significantly lower compared with WT male mice (Figure 3E). As for other cortical bone parameters, such as cortical bone area/total cortical area (Ct.Ar/Tt.Ar) and cortical thickness (Ct.Th), we found that *Phex*‐T1349C mice had significantly lower Ct.Ar/Tt.Ar (Figure 3F) and Ct.Th (Figure 3G) in both males and females compared to WT littermates.

**Figure 4:**
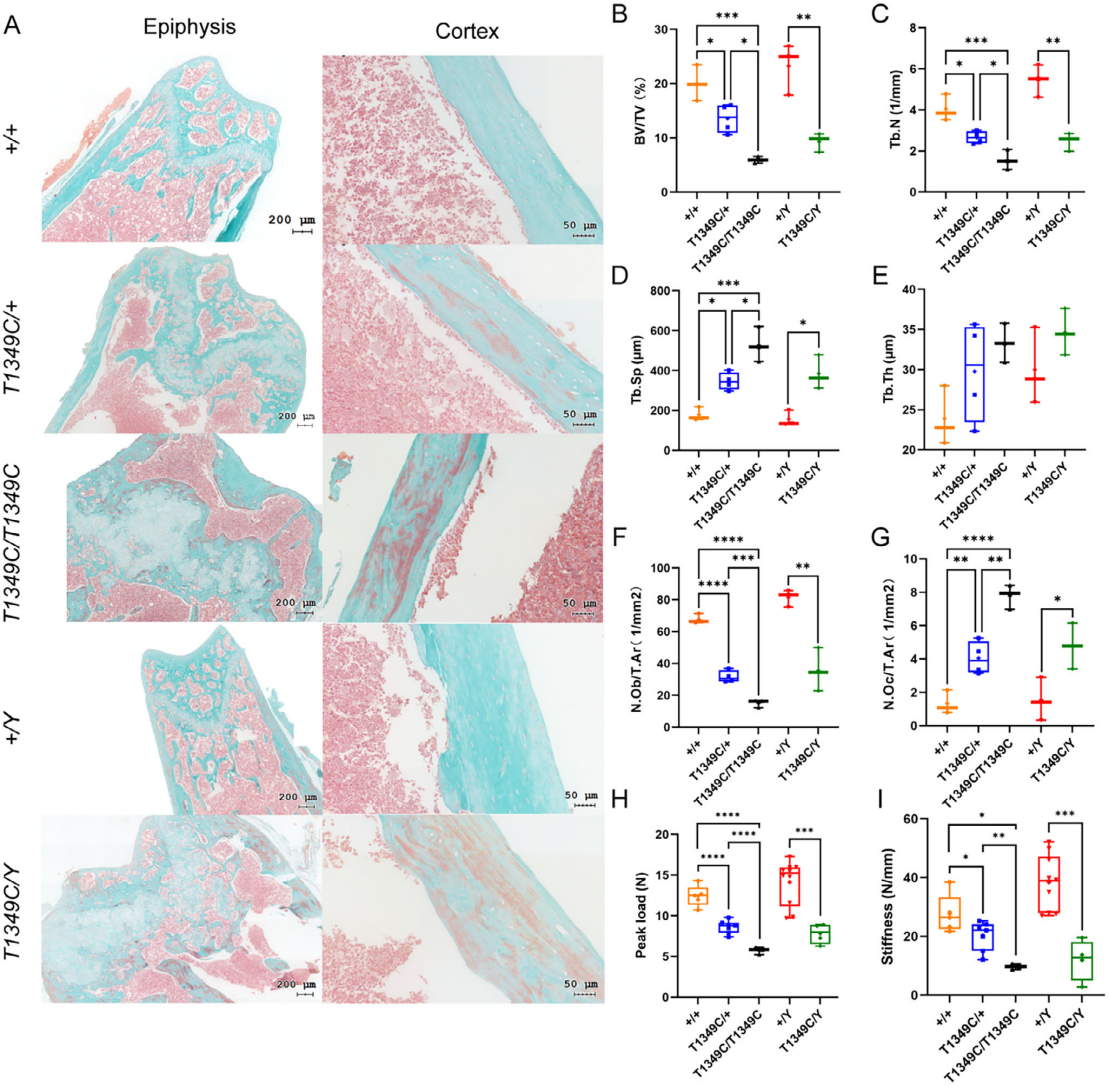
Bone histomorphometry analysis and three‐point bending test of the femurs of *Phex*‐T1349C mice and wild‐type (WT) littermates. Representative images of Goldner trichrome staining (A) demonstrated epiphyseal and cortical changes in WT and *Phex*‐T1349C mice. Bone histomorphometry analysis included B) BV/TV, C) trabecular number, D) trabecular separation, E) trabecular thickness, F) number osteoblast/tissue area, G) number osteoclast/tissue area. Peak load (H) and stiffness (I) in WT and *Phex*‐T1349C mice. For box‐and‐whiskers plots, the center line represents the median, the bounds of the box represent quartiles and the whiskers represent min to max (*N* = 3–7 each group), ^*^p < 0.05, ^**^ *p* <0.01, ^***^ *p*< 0.001, ^****^ *p* < 0.0001 by Student's *t*‐test or one‐way ANOVA for normally distributed continuous variables with homogeneous variance, or Mann Whitney test for non‐normally distributed continuous variables with nonhomogeneous variance.

Consistent with the skeletal abnormalities of XLHR, Goldner trichrome staining showed that femurs of *Phex*‐T1349C mice had obvious morphological deformities, including reduced trabeculae, under‐mineralized cortical bone and a significant disorganization of growth plate, consistent with typical features of rickets. The femoral growth plates of *Phex*‐T1349C mice were markedly abnormal with an enlarged proliferative zone and disorganization. In addition, noncalcified cartilage accumulated adjacent to the growth plate (**Figure** **4**A). The results of bone histomorphometric analysis were also in agreement with the tendencies of micro‐CT findings: *Phex*‐T1349C mice had significantly decreased BV/TV (Figure 4B), fewer trabeculae (Figure 4C) and greater trabecular separation (Figure 4D) in both males and females compared to WT littermates, whereas no difference in trabecular thickness was observed (Figure 4E). In addition, *Phex*‐T1349C mice demonstrated significantly decreased osteoblast number (Figure 4F) and increased osteoclast number (Figure 4G). It is worth noting that the skeletal abnormality of homozygous females was more severe than that of heterozygotes. The femurs of *Phex*‐T1349C mice showed significantly lower peak load and bone stiffness compared to WT littermates (Figure 4H,I).

### 2.5 The Expression of Bone and Kidney Markers in *Phex*‐T1349C Mice

**Figure 5:**
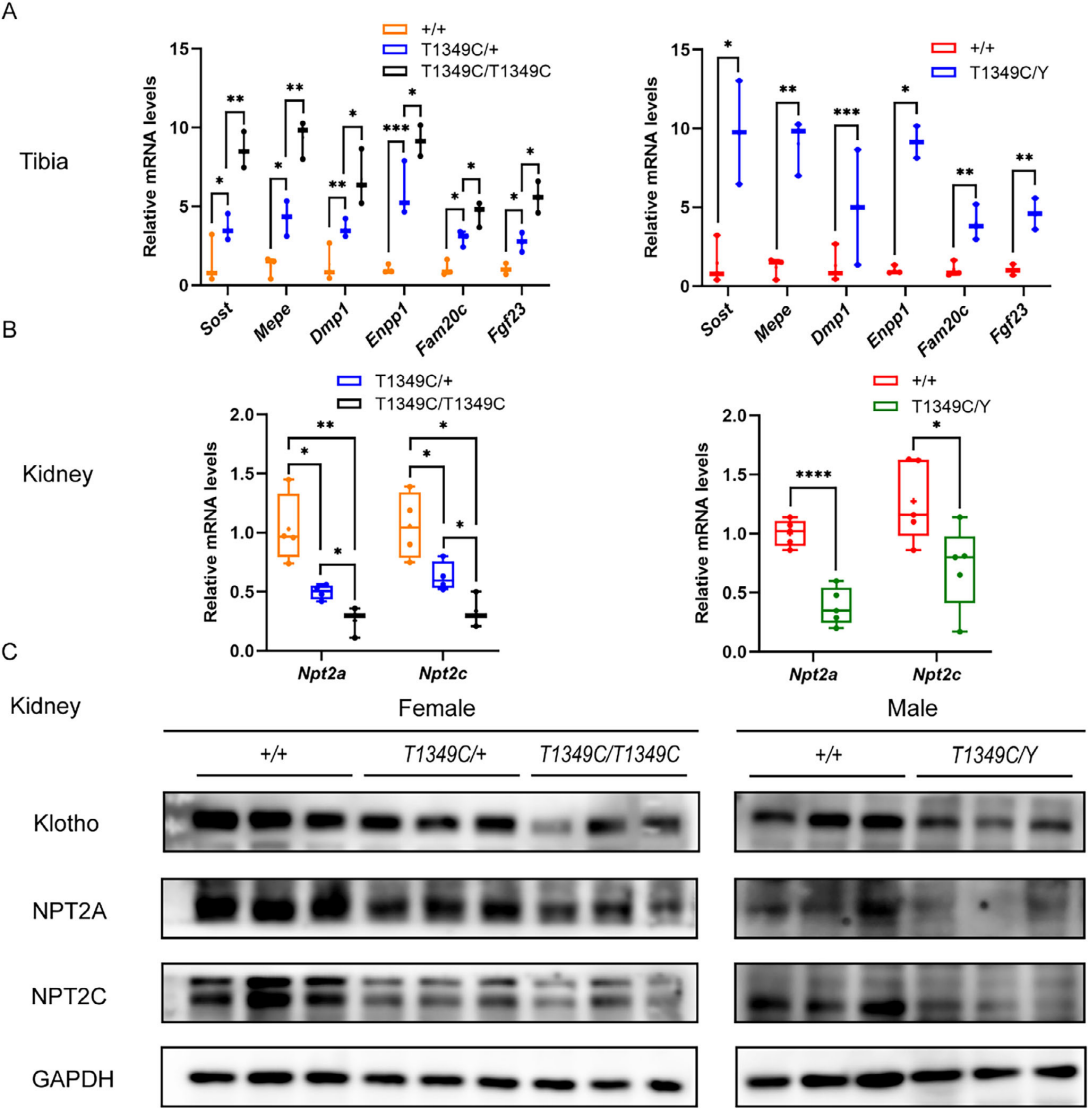
mRNA and protein expression levels of bone and kidney markers in tibiae and kidneys of *Phex*‐T1349C mice. A) mRNA levels of bone markers, including *Sost*, *Mepe*, *Dmp1*, *Enpp1*, *Fam20c*, and *Fgf23*, in tibiae of mice. B) mRNA levels of kidney markers, including *Npt2a* and *Npt2c*, in kidneys of mice. C) Representative western blots of Phex expression in lysates of tibiae of the indicated sex and strain of mice at 12 weeks of age. β‐actin antibody was used as a loading control. D) Representative western blots of the indicated proteins from the lysates of kidneys from the indicated sex and strain of mice at 12 weeks of age. GAPDH antibody was used as loading control. For box‐and‐whiskers plots, the center line represents the median, the bounds of the box represent quartiles and the whiskers represent min to max (*N* = 3–7 each group). ^*^ *p* < 0.05, ^**^ *p* < 0.01, ^***^ *p* < 0.001, ^****^ *p* < 0.0001 compared with sex‐matched WT mice. Student's *t*‐test or one‐way ANOVA for normally distributed continuous variables with homogeneous variance, or Mann Whitney test for non‐normally distributed continuous variables with nonhomogeneous variance.

Given that FGF23, the key phosphaturic factor in XLHR, is elevated in the serum of XLH mice, we examined the effect of the novel *Phex* variant on the mRNA expression level of *Fgf23*. In the tibia of *Phex*‐T1349C mice, *Fgf23* mRNA expression was significantly higher (**Figure** **5**A), consistent with the high levels of FGF23 in the serum of mutant mice. Some negative regulators of mineralization, such as *Sost, Mepe, Dmp1*, and *Enpp1*, were also increased in mutant mice (Figure 5A).

Furthermore, in the kidney, both the mRNA and protein expression of *Npt2a* and *Npt2c* were significantly decreased in mutant mice (Figure 5B,C). And the protein level of αKlotho was significantly decreased in the kidneys of *Phex*‐T1349C mice compared to age‐ and sex‐matched WT littermates (Figure 5C).

### 2.6 Phenotypic Rescue of *Phex*‐T1349C Mice by a Single Intramuscular Injection of MC.CMV‐FGF23 (180‐251) Without Apparent Adverse Effects

**Figure 6:**
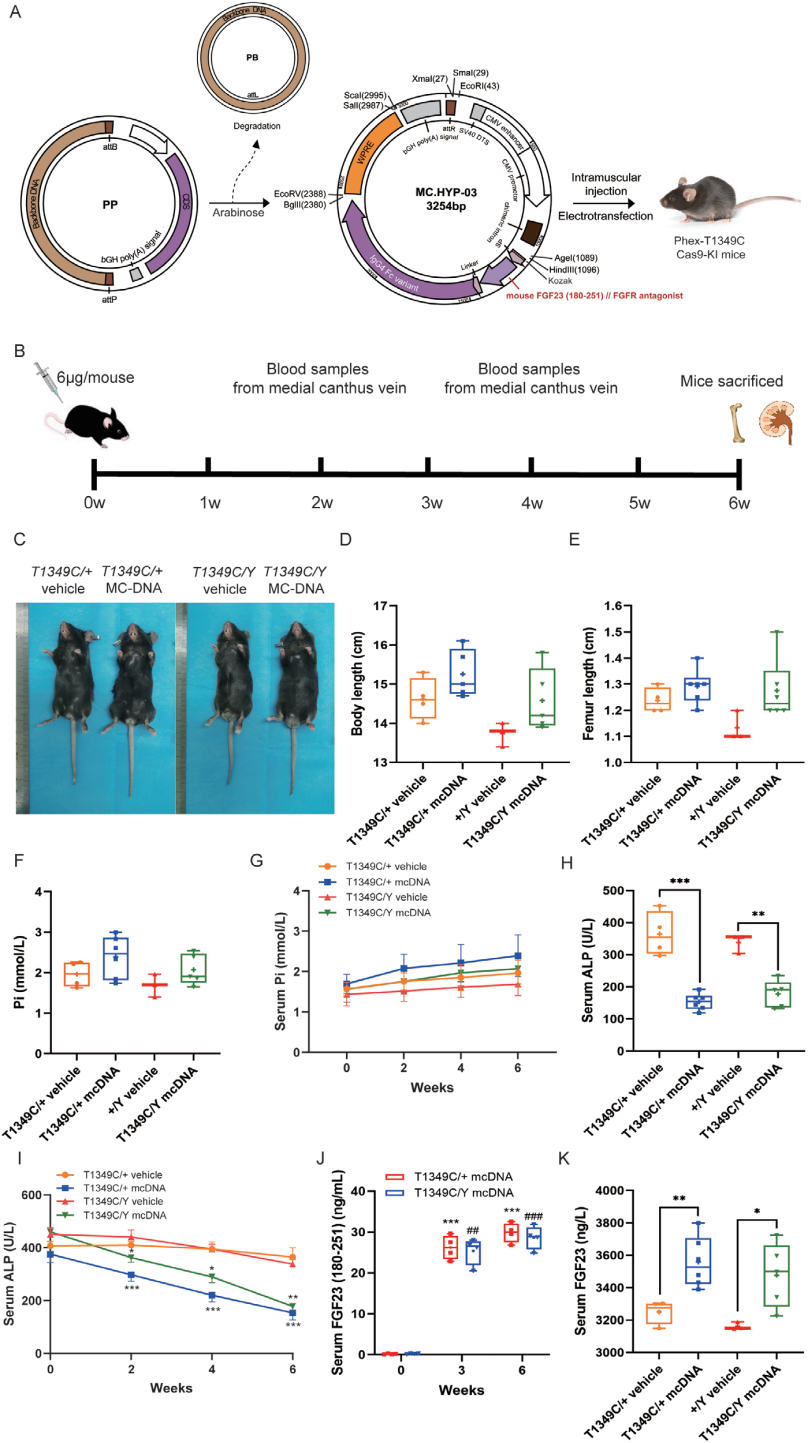
The construction of MC.CMV‐FGF23 (180‐251) and experimental schemes as well as the effects of MC‐DNA on growth parameters and phosphorus homeostasis in Phex‐T1349C mice. A) Flowchart demonstrating the production of CMV‐FGF23 (180‐251) using the established protocol. Briefly, the MC‐producing parental plasmid pMC.BESPX‐ FGF23 (180‐251) was constructed by placing the expression cassette including FGF23 (180‐251) under the control of the cytomegalovirus (CMV) promoter, into the MC making the plasmid pMC.BESPX between the AgeI and SalI sites. The parental plasmid was used to transform the MC‐producing *Escherichia coli* ZYCY10P3S2T. Overnight culture of the selected clone was processed to induce DNA recombination between the attB and attP sites mediated by PhiC31 expressed from the integrated inducible gene, resulting in the formation of two DNA circles, the MC and the plasmid backbone circle DNA. The plasmid backbone DNA was linearized by homing endonuclease I‐SceI also expressed from the inducible integrated gene, followed by degradation by bacterial exonucleases. Consequently, the MC remained as the only episomal circular DNA to be isolated from bacteria similar to regular plasmids. B) Timeline showed experimental procedure. Upticks show weeks in which blood was collected. 0w represented time at which 6 µg MC.CMV‐FGF23 (180‐251) was delivered into Phex‐T1349C mice via a single intramuscular injection. C) *Phex*‐T1349C mice (left: female, right: male) are smaller and have shorter tails than their WT littermates (left). D) Body length of age‐ and sex‐matched mutant mice treated with vehicle and MC‐DNA. E) Femoral length of age‐ and sex‐matched mutant mice treated with vehicle and MC‐DNA. Serum phosphorus (F,G), alkaline phosphatase (ALP) (H,I), FGF23(180‐251) (J), FGF23 (K) were examined during 6‐week treatment in *Phex*‐T1349C mice. Bars depict the mean ± SEM (*N* = 3–5 each group). For box‐and‐whiskers plots, the center line represents the median, the bounds of the box represent quartiles and the whiskers represent min to max. ^*^ *p* < 0.05, ^**^ *p* < 0.01, ^***^ *p* < 0.001, ^****^ *p* < 0.0001 represent significant difference between MC‐DNA treatment group and vehicle group, and ^##^ *p* < 0.01, ^###^ *p* < 0.001 represent significant difference from serum FGF23(180‐251) level of *Phex*‐T1349C hemizygous males at 0w by Student's *t*‐test for normally distributed continuous variables with homogeneous variance, or Mann Whitney test for non‐normally distributed continuous variables with nonhomogeneous variance.

As our newly established mouse model can fully reproduce the typical phenotypes of XLHR, we next wanted to use this mouse model to explore the potential therapeutic efficacy of a MC‐DNA approach. The isolated 72‐residue‐long C‐terminal tail of FGF23 (180‐251) has previously been shown to interfere with FGF23 signaling by competing with the full‐length ligand for binding to the FGFR‐Klotho complex, suggesting the possibility that it may serve as a potential therapeutic for the treatment of XLHR.^[^ ^36^ ^]^ Therefore, we constructed an MC‐DNA vector expressing MC.CMV‐FGF23 (180‐251) and delivered it to *Phex*‐T1349C mice at 4 weeks of age via a single intramuscular injection (**Figure** **6**A). Six weeks after the injection, we evaluated the phenotypic effects on the mice. The expression levels of FGF23 (180‐251) were significantly increased following MC‐DNA therapy (Figure 6J). We found that MC‐DNA treatment tended to slightly increase the body and femur length to correct the growth retardation of XLHR mice (Figure 6B–E). And it could also slightly increase serum Pi level, remarkably decrease serum ALP, and significantly increase serum FGF23 (Figure 6F–K).

**Figure 7:**
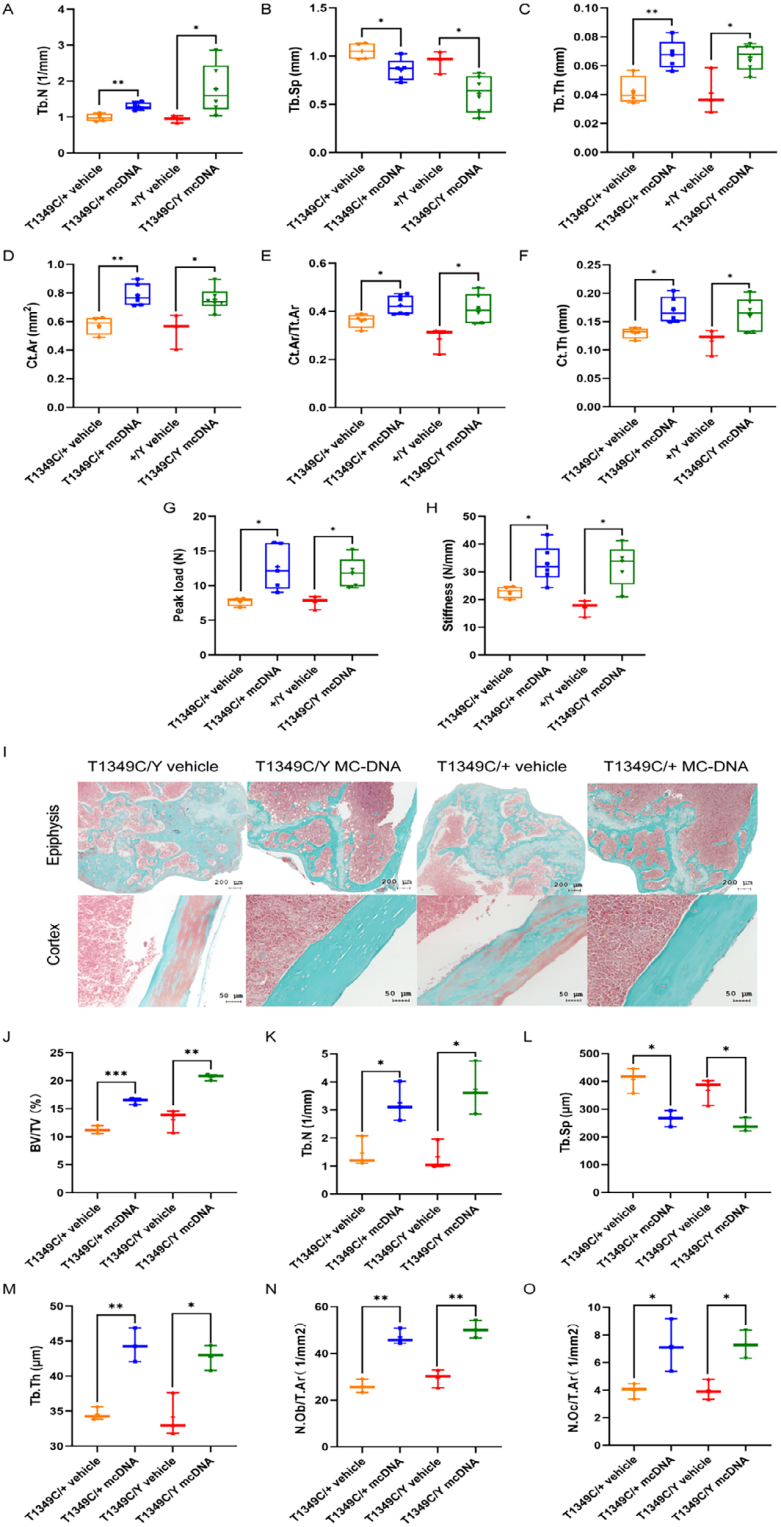
Micro‐CT analysis and bone histomorphometry of the femurs of *Phex*‐T1349C mice after MC.CMV‐FGF23 (180‐251) treatment. Trabecular bone parameters, including trabecular number (Tb.N) (A), trabecular separation (Tb.Sp) (B), and trabecular thickness (Tb.Th) (C), in female and male mice. And cortical bone parameters, including cortical bone area (Ct.Ar) (D), cortical bone area/ cortical total area (Ct.Ar/Tt.Ar) (E), and cortical thickness (Ct.Th) (F), in female and male mice treated with vehicle and MC‐DNA. Peak load (G) and stiffness (H) in treated female and male *Phex*‐T1349C mice. And representative images of Goldner trichrome staining (I) demonstrated epiphyseal and cortical bone changes in *Phex*‐T1349C mice treated with either vehicle or MC‐DNA. Bone histomorphometry analysis included J) BV/TV, K) trabecular number, L) trabecular separation, M) trabecular thickness, N) number osteoblast/tissue area, and O) number osteoclast/tissue area. For box‐and‐whiskers plots, the center line represents the median, the bounds of the box represent quartiles and the whiskers represent min to max (*N* = 3–5 each group). ^*^ *p* < 0.05, ^**^ *p* < 0.01 by Student's *t*‐test for normally distributed continuous variables with homogeneous variance, or Mann Whitney test for non‐normally distributed continuous variables with nonhomogeneous variance.

In addition, by micro‐CT analysis, we found that MC‐DNA therapy resulted in increased trabecular bone parameters (Tb.N and Tb.Th), while Tb.Sp was significantly reduced in female and male mice treated with either vehicle or MC‐DNA (**Figure** **7**A–C). In addition, cortical bone parameters, including Ct.Ar, Ct.Ar/Tt.Ar and Ct.Th, were remarkably higher in MC‐DNA treated female and male mice compared to the vehicle group (Figure 7D–F). Consistent with the changes in these bone parameters, the bone strength of the mutant mice was also significantly improved (Figure 7G,H). And the femoral growth plates of *Phex*‐T1349C mice were remarkably restored to an almost normal appearance, and the cortical bone mineralization was significantly improved (Figure 7I). Histomorphometric analysis showed that decreased BV/TV (Figure 7J), decreased trabecular number (Figure 7K), and increased trabecular separation (Figure 7L) in *Phex*‐T1349C mice were partially rescued with MC‐DNA treatment as well as increased trabecular thickness (Figure 7M). Moreover, *Phex*‐T1349C mice demonstrated significantly increased osteoblast number (Figure 7N) and osteoclast number (Figure 7O) following MC‐DNA treatment.

To evaluate the adverse effects of our MC‐DNA vector, we also measured aspartate aminotransferase (AST) and alanine aminotransferase (ALT), two markers of liver injury, and creatine (Cr) and blood urea nitrogen (BUN), two markers of kidney injury. No hepatotoxicity and nephrotoxicity was observed in treated mice 6 weeks after treatment (Figure S4A–D, Supporting Information). Hematoxylin and eosin staining also indicated that no liver or kidney damage or necrosis was observed (Figure S4E,F, Supporting Information).

### 2.7 The Association of MC.CMV‐FGF23 (180‐251) Treatment in *Phex*‐T1349C Mice with Molecular Mediators of Bone Homeostasis

**Figure 8:**
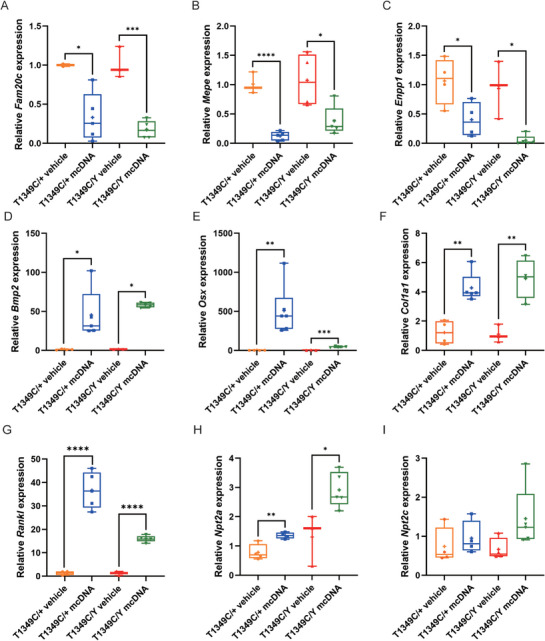
Expression level of key regulators of osteogenic differentiation and kidney markers in tibiae and kidneys of *Phex*‐T1349C mice after MC‐DNA treatment. The mRNA levels of some key regulators of osteogenic differentiation and bone mineralization, including *Fam20c* (A)*, Mepe* (B)*, Enpp1* (C)*, Bmp2* (D)*, Osx* (E)*, Col1a1* (F), *Rankl* (G) in tibiae of mice. mRNA levels of kidney markers, including *Npt2a* (H), *Npt2c* (I), in kidneys of mice. Tissues were taken from *Phex*‐T1349C mice treated with either vehicle or MC‐DNA. For box‐and‐whiskers plots, the center line represents the median, the bounds of the box represent quartiles and the whiskers represent min to max (*N* = 3–5 each group), ^*^ *p* < 0.05, ^**^ *p* < 0.01, ^***^ *p*< 0.001, ^****^ *p* < 0.0001 by Student's *t*‐test for normally distributed continuous variables with homogeneous variance, or Mann Whitney test for non‐normally distributed continuous variables with nonhomogeneous variance.

To elucidate the molecular changes that occur during the treatment with the MC.CMV‐FGF23 (180‐251) vector in *Phex*‐T1349C mice, we examined the expression of molecules involved in bone mineralization, osteoblast differentiation and osteoclast activity in the tibia, as well as the markers in the kidney. In the tibia, we found that the expression of a negative regulator of FGF23, *Fam20c*, was remarkably decreased (**Figure** **8**A), which could partially explain the high level of FGF23 in treated mice. And we evaluated two negative regulators of bone mineralization (*Mepe* and *Enpp1*) and found that both were decreased in MC‐DNA treated mice (Figure 8B,C). As for osteoblast differentiation, we examined the expression of *Bmp2*, which is a positive regulator of osteoblast differentiation through Smad signaling and regulates the expression of osteogenic genes, such as collagen type Ι (*Col1a1*). We found that the expression of *Bmp2* and its downstream osteogenic master transcription factor *Osterix* (*Osx*), as well as the osteogenic marker *Col1a1* in the tibia, were significantly increased after MC‐DNA treatment (Figure 8D,E,F). Furthermore, we explored the expression pattern of the osteoclast marker *Rankl* and found that the expression of *Rankl* was obviously increased in the tibiae of MC‐DNA treated mice (Figure 8G). These results were consistent with the histomorphometric analysis. In the kidney, we found that the mRNA expression of *Npt2a* and *Klotho* was significantly increased, while the expression of *Npt2c* was slightly increased in treated mice compared to the control group (Figure 8H,I).

## 3 Discussion

In this study, we first reported a Chinese XLHR pedigree carrying a novel *PHEX* variant (c.T1349C; p.L450P). Next, we used in vitro experiments to show that our newly discovered variant is pathogenic by impairing the expression, glycosylation, and catalytic activity of PHEX and facilitating the degradation of PHEX protein. Then we established a knock‐in XLHR mouse model by first introducing the c.T1349C variant into mice using CRISPR/Cas9 technology and our in vivo results demonstrated that typical XLHR clinical manifestations occurred in a gene‐dosage manner, including growth retardation, skeletal dysplasia (rickets/osteomalacia), and hypophosphatemia, together with elevated serum FGF23 and ALP. Finally, to the best of our knowledge, we also made the first reported attempt to perform MC‐DNA‐based therapy for XLHR in our *Phex*‐T1349C mice and found that the MC‐DNA vector increased blood phosphorus levels, decreased serum ALP levels and significantly improved bone mineralization of XLHR mice with no apparent adverse effects.

To date, there are eight XLHR mouse models with different mutations in the *Phex* gene, including Hyp (exon15‐22+3′UTR deletion), Gy (5′UTR+exon1‐3 deletion),^[^ ^37^ ^]^ Ska1(point mutation in exon 8),^[^ ^38^ ^]^ Hyp‐Duk(exon13‐14 deletion), Hyp‐2J (exons 15 deletion),^[^ ^39^ ^]^ Pug (defective glycosylation due to a Phe‐to‐Ser substitution at amino acid 80),^[^ ^40^ ^]^ Kbus/Idr (intragenic deletion of exon16‐18)^[^ ^41^ ^]^ and Jrt (point mutation in exon 14),^[^ ^42^ ^]^ three of which (Ska1, Pug, and Jrt) resulted from N‐ethyl‐N‐nitrosourea (ENU)‐induced mutagenesis. And they are further subdivided into *Phex*‐non‐specific (Hyp, Gy) and *Phex*‐specific models of XLHR (Hyp‐Duk, Hyp‐2J, Pug, Kbus/Idr and Jrt). Our model of XLHR is similar to the well‐studied Hyp mouse model: both have osteomalacic bone, hypophosphatemia, abnormal serum calcium and ALP levels, growth plate defects, and elevated FGF23 levels. And the severity of the phenotypes exhibited gene dose‐dependence. These features may provide a useful new tool for elucidating the multiple functions of PHEX. Finally, our new model will be a useful addition to the group of existing mouse models for the testing of new therapeutics for XLHR. A point worth mentioning is that in our *Phex*‐T1349C mice, the serum level of 1,25‐(OH)2D was significantly increased in heterozygous females and decreased in homozygous females, whereas that of hemizygous males were unchanged.^[^ ^43^ ^]^ We hypothesized that this might be related to the fact that the mutant protein retains partial enzymatic activity to maintain subnormal (partial) responsiveness of serum 1,25(OH)2D levels to phosphate depletion in *Phex*‐T1349C heterozygous females, but the function of Phex protein was predicted to be totally lost with a significant loss of negative feedback regulation in homozygous females. And this also indicates that the degree of *Phex* gene mutation is positively correlated with the phenotype. Further studies into specific mechanisms are worth considering in the future. It is also worth noting that we found the reproductive speed of our mouse model was faster than that of Hyp, Hyp‐Duk, and *Phex* knockout mice, indicating that our newly established mouse model is a potential candidate for future studies on XLHR.

Many studies are ongoing to address how *PHEX* loss‐of‐function leads to the biochemical and skeletal abnormalities observed in XLHR with multiple factors continuously being identified, among which FGF23 was a key regulator involved in a bone–kidney axis regulating phosphate homeostasis. FGF23 is synthesized by osteocytes (and osteoblasts) and mainly acts as a phosphaturic factor, an inhibitor of 1,25‐dihydroxyvitamin D, and a regulator of PTH synthesis and secretion,^[^ ^44^, ^45^, ^46^ ^]^ through MAPK (Erk1/2 and Akt) pathways in most tissues. Indeed, it inhibits the expression of type IIa and IIc sodium‐phosphate cotransporters (NPT2a and NPT2c) on the apical membrane of proximal tubular cells, thereby blocking tubular phosphate reabsorption.^[^ ^47^, ^48^ ^]^ It also inhibits the activity of 1α‐hydroxylase (CYP27B1) and stimulates the activity of 24‐hydroxylase (CYP24A1), leading to lower circulating levels of 1,25‐dihydroxyvitamin D.^[^ ^49^ ^]^ Thus, FGF23 is considered as a potential candidate target for the treatment of XLHR. Recently, a novel FGF23 antibody (Burosumab) has been approved for the treatment of patients with XLHR.^[^ ^11^, ^14^ ^]^ Blockade of FGF23 signaling by burosumab improved hypophosphatemia, 1,25(OH)2D deficiency and skeletal deformities in children and adults with XLHR. Consistent with these findings, in our study we showed that a naked MC‐DNA vector containing wild‐type human FGF23 (180‐251) driven by a CMV promoter [MC.CMV‐FGF23 (180‐251)], which competitively inhibits the binding of active FGF23 to its receptor, can partially correct the metabolic and phenotypic defects observed in *Phex*‐T1349C mice. The MC‐DNA vector lacks bacterial DNA sequences, which are eliminated by an inducible site‐specific recombinase present in the bacterial strain used for plasmid propagation. The absebce of bacterial sequences in the vector is thought to prevent transgene silencing, which is a major concern in gene therapy.^[^ ^50^ ^]^ And the intramuscular route of gene delivery is quite feasible in humans, making it a promising therapy for XLHR in the future.

As the MC‐DNA vector approach demonstrated good therapeutic effects in our study, we next attempted to explore the molecular mechanisms involved. The process of osteogenesis often starts with progenitor cells of osteoblastic lines, and then proceeds through three stages: cell differentiation, matrix maturation, and mineralization. We therefore studied osteogenesis from three main perspectives: osteoblast differentiation, osteoclast differentiation, and bone mineralization. Osteoblast differentiation is regulated by several cytokines, including bone morphogenetic proteins, transforming growth factor, Wnt, and Hedgehog.^[^ ^43^ ^]^ Among these, BMP2 (bone morphogenetic protein 2) is one of the most potent cytokines that promotes differentiation of mesenchymal cells into osteoblasts in vitro and induces bone formation in vivo. BMP2 exerts its osteogenic function by activating Smad signaling and further regulating transcription of osteogenic genes such as ALP, type I collagen (COL1A1), osteocalcin (OC), and bone sialoprotein (BSP).^[^ ^51^ ^]^ Osterix, also known as SP7, a member of the Sp1 transcription family, is upregulated by BMP2 to regulate the expression of many specific osteoblast differentiation markers including OC, COL1A1, and ALP during osteoblastic differentiation.^[^ ^52^, ^53^, ^54^ ^]^ As we found that the expression of *Bmp2*, *Osx*, and *Col1a1* was significantly increased after MC‐DNA treatment, we hypothesize that the MC.CMV‐FGF23 (180‐251) vector may promote osteogenesis by regulating the BMP2/Smad/Osterix pathway (Figure S5, Supporting Information). Whether other pathways also participate in the bone formation of treated mice still requires further investigation. Receptor activator of nuclear factor‐kappa B (NF‐κB) ligand (RANKL) is a cytokine that is essential for osteoclastogenesis, and it is expressed by osteoblasts as a membrane‐associated cytokine RANKL stimulates the differentiation of osteoclast, while OPG suppresses the differentiation of osteoclast.^[^ ^55^ ^]^ We found that the expression of *Rankl* was also increased following MC‐DNA treatment, also indicating that activated osteoclast differentiation was involved in the molecular mechanism of MC‐DNA therapy. The small integrin‐binding ligand N‐linked glycoprotein (SIBLING) family consists of osteopontin (OPN), bone sialoprotein (BSP), dentin matrix protein 1 (DMP1), dentin sialophosphoprotein (DSPP), and matrix extracellular phosphoglycoprotein (MEPE). These proteins share many structural characteristics and are primarily located in bone and dentin. Accumulating evidence has implicated the important role of SIBLING proteins in bone matrix mineralization.^[^ ^56^ ^]^ Thus, we also examined the expression of *Mepe* and *Enpp1* which are negative regulators of mineralization, and they were significantly decreased in the tibiae after MC‐DNA therapy. All of the above suggest that MC.CMV‐FGF23 (180‐251) vector rescued the skeletal abnormalities by promoting osteoblast differentiation, osteoclast differentiation, and bone mineralization. However, continued research into the mechanism underlying the bone remodeling caused by MC‐DNA therapy is required in the future.

Our study also has some limitations. We only examined the phenotypic changes in the mutant mice at a single endpoint. We will need to monitor the phenotypes of the treated mice at different time points after MC‐DNA administration over a longer period of time, as well as perform a dose–response study to identify the MC‐DNA dose for optimal therapeutic effects.

## 4 Conclusion

In summary, we conclude that the novel *PHEX* missense variant (c.T1349C; p.L450P) identified in an XLHR proband is pathogenic and that it leads to disease onset by reducing *PHEX* expression, facilitating PHEX protein degradation and preventing PHEX glycosylation. In addition, we established the first *Phex* Cas9‐KI mouse model that can faithfully reproduce the clinical phenotypes of XLHR. Moreover, we developed and tested a MC‐DNA‐based therapeutic approach that maintained transgene expression without apparent adverse effects in our newly constructed mouse model, suggesting that MC‐DNA may offer an improved safety profile over viral‐based vectors while also providing effective gene therapy for XLHR. However, similar to Burosumab, our MC‐DNA can only counteract the effects of FGF23 but cannot cure the disease from the root. New therapies that target *PHEX* are therefore more promising for curing XLHR.

## 5 Experimental Section

### Ethics Approval and Consent to Participate

This study has been approved by the Ethics Committee of Shandong Provincial Hospital affiliated to Shandong First Medical University (LCYJ:NO.2019‐147). The study protocol was in line with the Declaration of Helsinki (as revised in Brazil 2013). Informed consent was obtained from all individual participants included in the study and written informed consent was received from participants prior to inclusion in the study.

### Clinical Data Collection of the XLHR Proband

The proband was first admitted into Shandong Provincial Hospital for genu varum and waddling gait. His mother had similar symptoms. The proband received specific physical and laboratory examinations and the peripheral blood specimens were collected from each member for genetic analysis. Laboratory examinations included fasting serum phosphorus (Pi), calcium (Ca), ALP, parathyroid hormone (PTH), 25‐hydroxyvitamin D (25‐(OH)D) measured in the clinical laboratory of the hospital. Fibroblast growth factor 23 (FGF23) and α‐Klotho levels were measured using human FGF23 and soluble α‐Klotho ELISA kits (SenBeiJia Biological Technology Co., Ltd., China). Height was presented as standard deviation score (SDS) using standardized growth charts for Chinese children and adolescents aged 0 to 18 years.^[^ ^57^ ^]^

### DNA Extraction and Whole‐Exome Sequencing

Genomic DNA was isolated from peripheral blood leukocytes using the QIAamp DNA Mini Kit (Qiagen, Germany) following the manufacturer's instructions. Whole‐exome sequencing (WES) was performed on DNA from peripheral blood. After genomic DNA fragmentation, paired‐end adaptor ligation, amplification and purification, the human exons were captured by using the SeqCap EZ Med Exome Enrichment Kit (Roche NimbleGen, USA). The DNA library was generated by postcapture amplification and purification and then sequenced on the Illumina HiSeq sequencing platform. Sequence data alignment to the human genome reference (hg19) and variant‐calling were performed with NextGene V2.3.4 software to obtain the coverage and mean read depth of the target regions. The average coverage of the exome was > 100×, which permitted the examination of the target region with enough depth to exactly match >99% of the target exome. To ensure the accuracy of data analysis, mutations with low coverage in the target area would be filtered out.

Additionally, annotation information, including the conservation of nucleotide bases and amino acids, predictions of biological functions, the frequency in normal populations (Genome Aggregation Database (GenomAD), Trans‐Omics for Precision Medicine (TOPMED), the Exome Aggregation Consortium (ExAC)), and data from the Human Gene Mutation Database (HGMD), Clinvar and Online Mendelian Inheritance in Man (OMIM) databases, was performed by using NextGene V2.3.4 and in‐house scripts. A variant was recognized as a mutant when it was not found in dbSNP (http://www.ncbi.nlm.nih.gov/snp/), in the exome variant server (http://evs.gs.washington.edu/EVS/), in the ensemble database and in 500 Chinese controls, or alternatively, the allele frequency was found to be less than 0.001 in the database. Pathogenic variants were determined according to the Standards and Guidelines for the Interpretation of Sequence Variants published by American College of Medical Genetics and Genomics (ACMG) in 2015 with the Human Genome Variation Society (HGVS) nomenclature.

WES was used to detect candidate variants. When the detected pathogenic or suspected pathogenic variants was identified, the laboratory verified it by Sanger sequencing and ensured that the coverage of the gene coding sequence reached 100%. Using Primer3 version 1.1.4 (http://www.sourceforge.net) and GeneDistiller 2014 (http://www.genedistiller.org/), tagged sequencing primers of *PHEX* were designed. Polymerase chain reaction (PCR) was performed in a 50 µL system including 4 µL genomic DNA, 1 µL forward and reverse primers, 5 µL 10 × PCR buffer, 4 µL dNTPs, and 0.3 µL Taq Hot Start (Takara Bio, Ohtsu, Japan). The PCR conditions were as follows: an initial denaturation step (95 °C for 5 min), followed by 40 cycles of denaturation (95 °C for 30 s), annealing (65 °C for 30 s), and elongation (72 °C for 30 s). Amplicons were sequenced using an ABI 3730 system (Applied Biosystems, Foster City, CA, USA), and sequence analysis was performed using the autoassembler software Chromas 2.6.6 (Technelysium Pty Ltd).

### Bioinformatic Analysis

To test whether the mutation was benign or malignant, software PolyPhen‐2 (http://genetics.bwh.harvard.edu/pph2/) and Mutation Taster (http://www.mutationtaster.org/) were performed to predict potential effect. Multiple sequence alignment was performed by using Clustal Omega (https://www.ebi.ac.uk/Tools/msa/clustalo/). Tertiary structure model of the wild type and mutant PHEX was predicted from ITASSER (https://zhanglab.ccmb.med.umich.edu/I‐TASSER/). All the models were presented on the software of PyMOL (version 1.3).

### Plasmid Construction of PHEX

Full length of the major transcript of human PHEX (transcript ID: NM_000493.4) was synthesized and cloned into the transient overexpression vector GV141 (GeneChem, China), using the restriction enzymes XhoI and BamHI (NEB, USA). Each mutant *PHEX* was created by the Quickchange mutagenesis kit (Stratagene, La Jolla, USA) according to the manufacturer's instructions. The sequences of the plasmids were confirmed by Sanger sequencing.

To test the activity of PHEX enzyme both in WT and PHEX substitutions groups, a plasmid with soluble and secreted form of PHEX, also known as secPHEX, was constructed as previously described.^[^ ^35^ ^]^ After the same method of transfection in HEK293 cells described above, whole cell lysates and culture medium were collected for cell PHEX and secPHEX extraction. Cell PHEX extraction was the same as previously mentioned, the method of secPHEX extraction was performed according to the previous study,^[^ ^58^ ^]^ purified WT and mutant secPHEX were used for activity analysis.

### Cell Culture and Transfection

HEK293 cells (National Collection of Authenticated Cell Cultures, Shanghai, China) were grown in Dulbecco's Modified Eagle's Medium (Gibco BRL, Gaithersburg, MD, USA) containing 10% fetal bovine serum at 37 °C in 5% CO_2_ and 95% air. Cells were seeded in 6‐well plate prior to transfection and when they reached about 70% confluent, they were transfected with constructed PHEX overexpression vector or empty vector using Lipofectamine 3000 Transfection Kit (Invitrogen, USA). Transfection was performed for 8–10 h with 2 µg plasmid per well, and then they were each transiently transfected into HEK293 cells using Lipofectamine3000 (L3000‐015, Invitrogen). The whole cell lysates were collected for protein extraction 48 h after transfection.

### mRNA Isolation and Real‐Time Quantitative PCR

Total RNA was extracted using TRIzol reagent (Takara) according to the manufacturer's instructions. Next, reverse transcription reagent (Takara) was used to translate 1 µg of RNA into complementary DNA. Real‐time PCR was carried out with the LC480 system (Roche) using SYBR Green (Yeason). The primers used in the experiment are listed in Table S1 (Supporting Information). β‐Actin or Gapdh was used as an internal reference for each sample. Data was calculated by the 2^−ΔΔCT^ method, and expressed as the fold change relative to the control group for each experimental group.

### Immunoblot Analysis

Cells or tissues were lysed in radioimmunoprecipitation assay (RIPA) buffer supplemented with protease and phosphatase inhibitors. Protein lysates (cell 40 µg, tissue 60 µg) were separated using 10% SDS‐PAGE and transferred onto polyvinylidene fluoride membranes (Millipore). The membranes were blocked with TBST containing 5% slim milk for 1 h at room temperature and then incubated with primary antibodies against PHEX (1:1000, ProteinTech), Klotho (1:1000, ProteinTech), SLC34A1 (1:1000, Novus biologicals), SLC34A3 (1:1000, Abcam), Actin (1:7500, ProteinTech) and GAPDH (1:7500, ProteinTech) overnight at 4 °C, after which they were incubated with secondary antibodies for 1 h at room temperature. The membranes were incubated with HRP‐labeled secondary antibodies at room temperature for 1 h, and Immobilon Western HRP Substrate Peroxide Solution (Millipore, USA) was used for membrane development.

### Immunofluorescence Assay

Forty‐eight hours after transfection, cells were grown on glass coverslips and cell culture dishes were fixed with 4% paraformaldehyde, permeabilized with 0.5% Triton X‐100, and blocked for 1 h in 5% FBS. Immunostaining was accomplished with anti‐PHEX (1:200; Biorbyt, Cambridge, UK) over night at 4 °C. Species specific Alexa Fluor 488 secondary antibodies (Invitrogen, Waltham, MA) were used at 1:1000 at room temperature for 1 h. Nuclei were visualized by DAPI (4′6‐diamidino‐2‐phenylindole, blue). Protein localization was observed by fluorescence microscopy (Carl Zeiss, Germany).

### Deglycosylation Analysis

For endo H digestion, WT and the altered PHEX in the whole cell extracts were boiled for 10 min in 1× Glycoprotein Denaturing buffer and incubated with endo H for 1 h at 37 °C according to the manufacturer's recommendations (New England Biolabs). For the PNGase F digestion, the cell extracts were first incubated with 1×GlycoBuffer and mixed with PNGase F gently, then reacted with PNGase F at 37 °C for 5 h according to the manufacturer's recommendations (New England Biolabs). Digestion products were fractionated on SDS‐PAGE and subjected to immunoblot analysis as described above.

### Enzymic Activity of WT and Mutant secPHEX

SecPHEX activity in different groups were assayed in 0.01 m Bis/Tris buffer (pH 5.5) with 150 × 10^−3^ m NaCl routinely used to test enzymic activity. Purified secPHEX both in WT and mutant groups were quantified by western blot. Abz‐GFSDYK(Dnp)‐OH (30 × 10^−6^ m), a peptide containing a putative secPHEX cleavage site, was used as a substrate to interact with secPHEX in 0.01 m Bis/Tris buffer at 37 °C. After monitoring the reaction in the kinetic mode using a SpectraMax Gemini EM (Molecular Devices) for 60 min, the fluorescence was measured at *λ* (emission) = 420 nm and *λ* (excitation) = 320 nm.

### Phex Cas9‐KI Mice Generation

All animal experimental protocols were approved by the Animal Care Committee of Shandong Provincial Hospital (No.2022‐062). Animals with novel missense variant in the *Phex* gene (Hemizygous males: T1349C/Y; heterozygous females: T1349C/+; homozygous females: T1349C/T1349C) were generated from Gempharmatech Corp. in China via CRISPR/Cas9 technology with a C57BL/6J background. The breeding strategy generated heterozygous, homozygous, and WT females as well as hemizygous and WT males. Mice were caged in groups of 3 to 5, maintained on a 12‐h dark/light cycle, and were provided standard Global 18% protein rodent chow diet (Beijing Keaoxieli Feed Co. Ltd; 1–1.8% Ca, 0.6–1.2% Pi) and water ad libitum. Animals were housed under standardized conditions with ad libitum access to food and water to control for nutritional variables. The analysis of the phenotypes of *Phex*‐T1349C mice at 12 weeks of age was performed. As for the treatment part of the study, the mutant mice received treatment at 4 weeks of age and were monitored for 6 weeks.

Blood samples were collected by eyeball extirpation, then centrifuged to collect serum and stored at −80 °C for further biochemical analysis. Left femurs were collected, wrapped in saline‐soaked gauze, and then frozen for three‐point bending mechanical test. Right femurs were fixed in paraformaldehyde and used for micro‐CT analysis and bone histology. Right kidneys were also fixed in paraformaldehyde for histomorphology. Both tibiae and left kidneys were immediately frozen in liquid nitrogen for real‐time PCR and western blot (WB) analysis. A small piece of each liver was cut and fixed in paraformaldehyde for histomorphology and the rest was frozen in liquid nitrogen for real‐time PCR and WB analysis.

### Minicircle DNA Construction and Administration

MC.CMV‐FGF23 (180‐251) was generated following the protocol of the ZYCY10P3S2T system with modifications (Syno Minicircle Biotechnology Co. Ltd., China).^[^ ^28^ ^]^ Briefly, a seed culture was prepared using one transformed colony in 20 mL of TB with kanamycin and cultured at 37 °C with shaking at 250 rpm until reaching the logarithmic phase (OD_A600_ = 2.0). Subsequently, an expanded culture was composed by adding 4 mL of the seed culture to 200 mL of TB containing kanamycin in a 500 mL flask. The culture was incubated at 37 °C with shaking at 250 rpm for 10 h to the stationary phase. At this point, the MC formation reaction was initiated by adding equal volumes of induction mix comprising 200 mL of LB medium and 40 µL of 20% filtered l‐arabinose that was adjusted to pH 7.0 using an appropriate volume of sodium hydroxide. The reaction continued with shaking at 250 rpm at 30 °C for an additional 5 h before being processed to isolate the MC‐DNA from bacterial lysates using a commercially available affinity column (Qiagen). The generated MC‐DNAs were also adjusted to 1 µg/µL using TE buffer.

MC.CMV‐FGF23 (180‐251) or MC.CMV‐Luciferase was administered by single intramuscular injection at gastrocnemius. *Phex*‐T1349C mice were randomly divided into two groups: the MC.CMV‐Luciferase vehicle group and the MC.CMV‐FGF23 (180‐251) treatment group. The mice received one i.m. dose of 3 µg MC‐DNA in 50 mL PBS each, into the gastrocnemius of both legs (6 µg MC‐DNA per mouse). The rationale for selecting 6 µg dose referred to a previously published literature.^[^ ^59^ ^]^ After 6 weeks, the phenotypical changes of mice were evaluated. All mice were euthanized by tribromoethyl alcohol overdose.

### Genotyping of Mice

DNA was extracted from tail tissue at 3 weeks of age using a TIANamp Genomic DNA Kit following standard procedures (TIANGEN, China). Then PCR was performed as follows: 95 °C for 5 min, 20 cycles of 98 °C for 30 s, 65 °C (‐0.5 °C/cycle) for 30 s, and 72 °C for 45 s, 20 cycles of 98 °C for 30 s, 55 °C for 30 s, and 72 °C for 45 s, and a final extension of 72 °C for 5 min. The products were then analyzed on a 3730xl DNA analyzer (ABI, USA) to determine whether the mutation site had been inherited. The specific mutation was identified by direct sequencing of *Phex* exons from amplified genomic DNA.

### Faxitron X‐Ray

X‐ray pictures of the whole mice were taken using a DR uDR588i from United Imaging Corp. in China. X‐ray images were taken under constant conditions (50 kV, 40 ms). Analysis of body and femur lengths were determined from the DICOM files using the RadiAnt DICOM Viewer software (Poznan, Poland).

### Biochemistry Analysis

Serum Pi, Ca, creatinine, BUN, AST, ALT, and ALP levels were measured using an automatic biochemical analyzer (BS‐830, Mindray, China). Serum fibroblast growth factor 23 (FGF23), α‐Klotho, parathyroid hormone (PTH), 1,25‐(OH)_2_D, and FGF23(180‐251) levels were measured using mouse FGF23, soluble α‐Klotho, PTH, 1,25‐(OH)_2_D elisa kits (SenBeiJia Biological Technology Co., Ltd., China) and human IgG Fc elisa kit (Novus Biologicals, USA) following the manufactures instruction. Twenty‐four urine samples were collected and renal phosphate excretion was determined. Renal phosphate excretion is expressed as fractional phosphate excretion (FPE_PO4_). FPE_PO4_ is calculated by urine phosphate concentration times serum creatinine concentration divided by serum phosphate concentration times urine creatinine concentration.

### Micro‐CT (µCT) Analysis

Right femurs of the mice were stored in paraformaldehyde before scanned. Scanning parameters were 70 kVp and 114 µA, with a 250 ms integration time and a 15.6 µm isotropic voxel size (vivaCT60, Scanco Medical). Cortical geometry was measured in the middle 100 slices of the femoral diaphysis. Primary cortical parameters included cortical area (Ct.Ar), total area (Tt.Ar), and cortical thickness (Ct.Th). Trabecular bone architecture was analyzed from the distal 120 slices of the total femoral length to the distal growth plate. Primary trabecular parameters mainly included trabecular number (Tb.N), trabecular thickness (Tb.Th), and trabecular spacing (Tb.Sp). All parameters are reported using conventional nomenclature according to ASBMR standards. Group sample sizes for all µCT analyses ranged from *n* = 3 to 7 mice.

### Mechanical Test

Left femurs of the mice were thawed in saline prior to 3‐point bending. Femurs were loaded to fracture in the anterior‐posterior direction. A lower support span length of 7 mm was used for mice. A load rate of 0.1 mm s^−1^ and a preload of ≈0.5 N were applied to each bone to prevent shifting during testing (3230 SERIES III, WATERS Corp.). Load–displacement curves were used to calculate the peak load and bending stiffness. Group sizes for mechanical testing ranged from *n* = 3 to 7 mice.

### Bone Histomorphometry

Following µCT scanning, the femur was dehydrated through a series of graded alcohol solutions and then embedded in paraffins. Longitudinal sections were cut to at a 5 µm thickness and mounted onto glass slides. Slides were stained with Goldner's trichrome. Structural histomorphometric parameters were quantified using the Osteomeasure System (R&M Biometrics, Nashville, TN) according to ASBMR standards. Bone volume per trabecular volume (BV/TV [%]), trabecular number (Tb.N. [1 mm^−^]), trabecular thickness (Tb.Th. [µm]), trabecular separation (Tb.Sp. [µm]), number osteoblast/Tissue area (N.Ob/T.Ar [mm^−2^]), and osteoclast/Tissue area (N.Oc/T.Ar [mm^−2^]) were measured. Osteoblasts were identified as cuboidal cells lining the trabecular bone. Osteoclasts were identified as multinucleated cells with more than three nuclei on the trabecular bone surface. Group sample sizes for histological analysis ranged from *n* = 3 to 4 mice.

### Statistical Analysis

Statistical analysis was performed using GraphPad Prism software (version10.4.1). The Kolmogorov‐Smirnov test was used to determine the distribution of continuous variables. Continuous variables with normal distribution were presented as mean ± SEM or box and whisker plots with indication of median and interquartile ranges showing all data points and compared using two‐tailed independent samples Student's *t*‐test or one‐way ANOVA followed by Tukey's post‐hoc test for multiple comparisons. Variables with non‐normal distribution were presented as box and whisker plots with indication of median and interquartile ranges showing all data points and compared using Mann‐Whitney U‐test. Sample sizes (*n*) for each statistical analysis are specified in respective figure legends. A significance level of *α* = 0.05 was used for all tests, and results were considered statistically significant when *p* < 0.05.

## Conflict of Interest

The authors declare no conflict of interest.

## Author Contributions

J.Z. and C.X. designed and supervised the study as well as mainly revised the manuscript. H.W. performed the most experiments and wrote the manuscript. Y.W. and Y.Y. were responsible for clinical data collection. W.Z., X.C., B.X., R.Q., J.X., and S.X. assisted H.W. to perform the experiments. P.C. and Z.C. contributed to MC‐DNA production. S.W., Y.S., Z.L., M.S. provided support to finish some special experiments. Y.W., W.X., and L.G. helped to review the manuscript. All authors approved the final manuscript.

## Supporting information



Supporting Information

Supporting Information

Supporting Information

## Data Availability

The data that support the findings of this study are available from the corresponding author upon reasonable request.
