# Supplementary material for: Treatment with Minicircle DNA Expressing a FGF23 Fragment in a Clinically relevant Mouse Model of X‐Linked Hypophosphatemic Rickets
Source: Adv Sci (Weinh). 2025 Oct 31;13(4):e08870. doi: 10.1002/advs.202508870 (PMC12822395; doi:10.1002/advs.202508870)
Supplement: Supplementary file 1 — Supporting Information [file ADVS-13-e08870-s003.docx]

Supporting Information

Treatment with Minicircle DNA Expressing a FGF23 Fragment in a Clinically-relevant Mouse Model of X-linked Hypophosphatemic Rickets

Huixiao Wu, Wanyi Zhao, Xinyu Chen, Yanzhou Wang, Shuoshuo Wei, Bo Xiang, Yingzhou Shi, Zongyue Li, Yangyang Yao, Jin Xie, Renyuan Qiu, Meng Shu, Shuo Xu, Ping Chen, Zhiying Chen, Yingjie Wu, Weibo Xia, Ling Gao, Yongfeng Song, Jiajun Zhao* and Chao Xu*

**Supplement Table 1.** **Laboratory parameters of the patient at the first clinical evaluation**

| **Parameters** | **Results** | **Reference range** |
| --- | --- | --- |
| Serum calcium (mmol/L) | 2.35 | 2.2-2.7 |
| Serum phosphate (mmol/L) | 0.67 | 0.85-1.51 |
| 25-hydroxyvitamin D (ng/mL) | 10.47 | 15-100 |
| Parathyroid hormone (pg/mL) | 129.7 | 15-65 |
| Alkaline phosphatase (U/L) | 1085 | 45-125 |
| Creatinine (μmol/L) | 34.91 | 40-135 |
| FGF23 (ng/L) | 1432.55 | 235.3±87.2 |
| α-Klotho (ng/L) | 15864 | 876.5±312.4 |

**Supplement Table 2.** **Primers used for quantitative PCR analysis**

| **Gene** | **Species** | **Forward or reverse primers** | **Primer Sequences** |
| --- | --- | --- | --- |
| *PHEX* | Human | F | 5’-GTACTGCCTGAAGCCAGAATGC-3’ |
|  |  | R | 5’-TCCAGCCATCACAAGCGAACC-3’ |
| *Fgf23* | Mouse | F | 5’-TGGACGCTGGAGAATGGCTATG-3’ |
|  |  | R | 5’-CCGTGGGCGAACAGTGTAGAAA-3’ |
| *Fam20c* | Mouse | F | 5’-GCCAAGTTGTTTGAGCACCC-3’ |
|  |  | R | 5’-TTCATGCGGCCAGTCAGGAT-3’ |
| *Sost* | Mouse | F | 5’-GTGCCTCATCTGCCTACTTGT-3’ |
|  |  | R | 5’-CCGACACATCTTTGGCGT-3’ |
| *Dmp1* | Mouse | F | 5’-AGTGAGGAGGACAGCCTGAA-3’ |
|  |  | R | 5’-GAGGCTCTCGTTGGACTCAC-3’ |
| *Enpp1* | Mouse | F | 5’-CTGGTTTTGTCAGTATGTGTGCT-3’ |
|  |  | R | 5’-CTCACCGCACCTGAATTTGTT-3’ |
| *Mepe* | Mouse | F | 5’-GTCTGTTGGACTGCTCCTCTT-3’ |
|  |  | R | 5’-CACCGTGGGATCAGGATACA-3’ |
| *Cyp24a1* | Mouse | F | 5’-CTGCCCCATTGACAAAAGGC-3’ |
|  |  | R | 5’-CTCACCGTCGGTCATCAGC-3’ |
| *Cyp27b1* | Mouse | F | 5’-TCCTGGCTGAACTCTTCTGC-3’ |
|  |  | R | 5’-GGCAACGTAAACTGTGCGAA-3’ |
| *Npt2a* | Mouse | F | 5’-TGCCTCTGATGCTGGCTTTC-3’ |
|  |  | R | 5’-GATAGGATGGCATTGTCCTTGAA-3’ |
| *Npt2c* | Mouse | F | 5’-CAACCCTACTCTGGATGCCTT-3’ |
|  |  | R | 5’-CTCTTTCAGTTGGTCAGCGTT-3’ |
| *Bmp2* | Mouse | F | 5’-AGCCAAACACAAACAGCGGAAG-3’ |
|  |  | R | 5’-GGAGTTCAGGTGGTCAGCAAGG-3’ |
| *Osterix* | Mouse | F | 5’-TACGGCAAGGCTTCGCATCTG-3’ |
|  |  | R | 5’-GCTCAAGTGGTCGCTTCTGGTA-3’ |
| *Col1a1* | Mouse | F | 5’-GGTCCTCGTGGTGCTGCT-3’ |
|  |  | R | 5’-ACCTTTGCCCCCTTCTTTG-3’ |
| *Alp* | Mouse | F | 5’-CACGGCGTCCATGAGCAGAAC-3’ |
|  |  | R | 5’-CAGGCACAGTGGTCAAGGTTGG-3’ |
| *Rankl* | Mouse | F | 5’-CACCATCAGCTGAAGATAGT-3’ |
|  |  | R | 5’-CCAAGATCTCTAACATGACG-3’ |
| *Opg* | Mouse | F | 5’-ATCCAAGACATTGACCTCTGTG-3’ |
|  |  | R | 5’-CTGTGGTGAGGTTCGAGTGG-3’ |

**Supplement Table 3. Antibodies used for western blot and immunofluorescence**

| **Antibody** | **Application** | **Company** | **Catalog number** | **Dilution factor** |
| --- | --- | --- | --- | --- |
| PHEX (Human) | WB | Proteintech | 66934-1-Ig | 1:1000 |
| PHEX (Mouse) | WB | Abcam | ab13667 | 1:1000 |
| α-Klotho | WB | Proteintech | 67331-1-Ig | 1:1000 |
| NPT2A | WB | Novus Biologicals | NBP2-13328 | 1:1000 |
| NPT2C | WB | Abcam | ab155986 | 1:1000 |
| GAPDH | WB | Proteintech | 60004-1-Ig | 1:7500 |
| β-Actin | WB | Proteintech | 66009-1-Ig | 1:7500 |
| Peroxidase-AffiniPure Goat Anti-Rabbit IgG (H+L) | WB | Jackson ImmunoResearch | 111-035-003 | 1:5000 |
| Peroxidase-AffiniPure Goat Anti-Mouse IgG (H+L) | WB | Jackson ImmunoResearch | 115-035-003 | 1:5000 |
| PHEX antibody (Human) | IF | Biobyt | orb422843 | 1:200 |
| Donkey anti-rabbit Alexa 488 | IF | Invitrogen | A32790TR | 1:1000 |


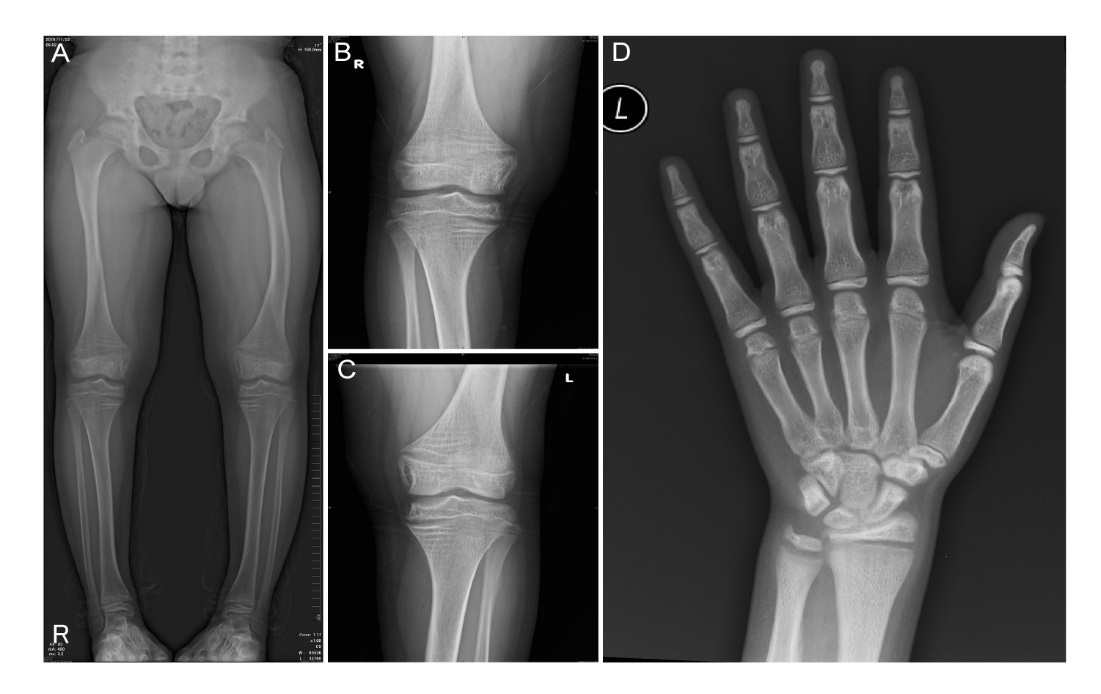


**Supplement Figure 1**. **Clinical images of the patient with XLHR.** (A) Radiograph of bilateral lower limb showed genu varum deformity. Radiographs of right (B) and left (C) knee joint showed brush-shaped epiphyses and bilateral metaphyseal expanding of the proximal tibia and distal femur. (D) Left hand and wrist X-ray also identified cup- and brush-shaped epiphyses accompanied with a delayed bone age.


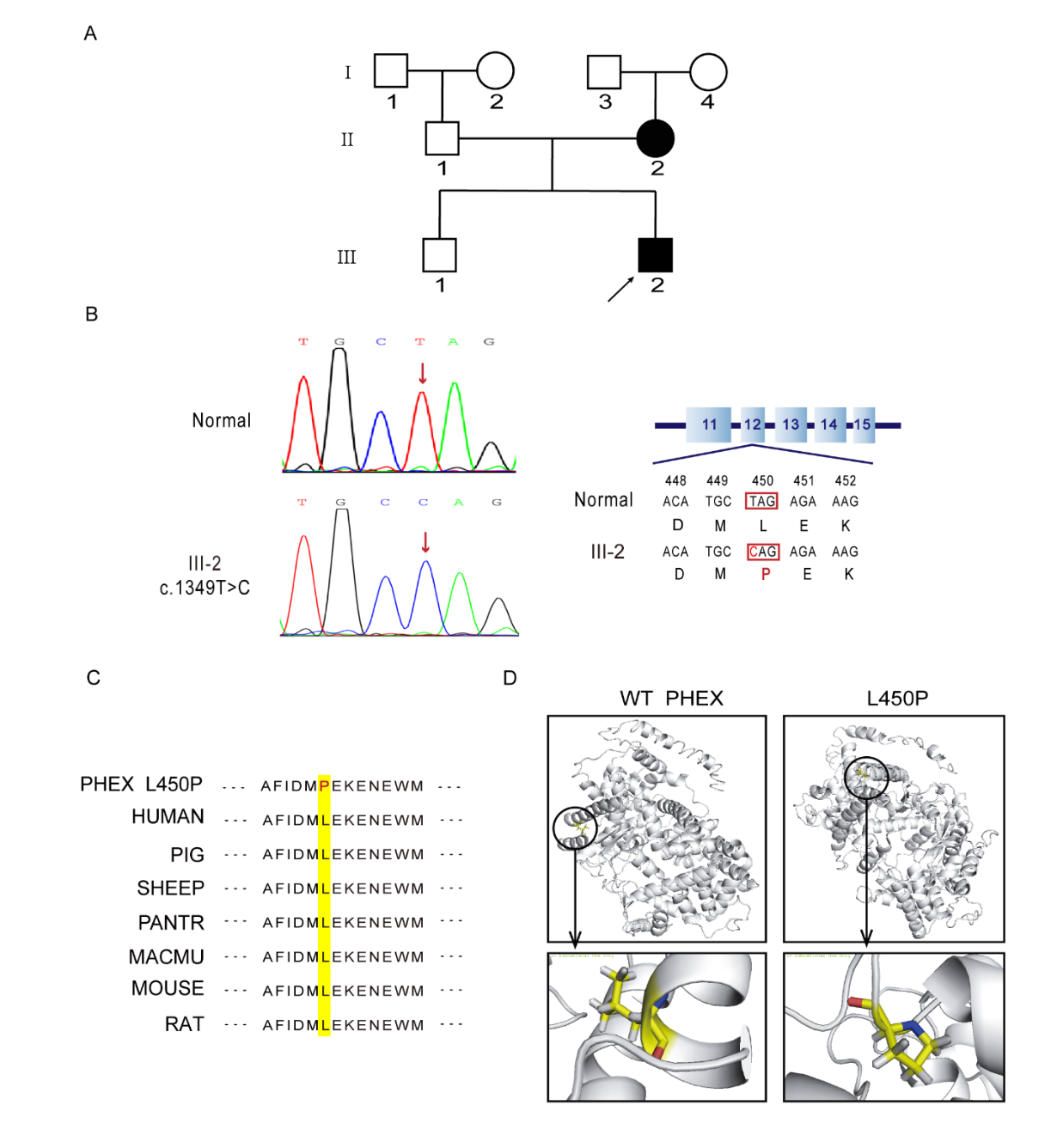


**Supplement Figure 2**. **The pedigree of a Chinese family with XLHR and bioinformatic analysis of the novel *PHEX* variant (c.1349T>C).** (A) The pedigree of a Chinese family with XLHR. Males and females are indicated by squares and circles, respectively. Affected individuals are represented by filled symbols. The proband is represented by the arrow. (B) Partial DNA sequence of the mutation site (c.1349T>C) in the *PHEX* gene and the amino acid sequence of p.450L>P variant in our proband. Altered nucleotides and amino acids are marked in red. Red boxes represent affected codons. (C) Conservation analysis of the c.1349T>C variant via multiple sequence alignment. Changed amino acids are marked in red. (D) Protein structure prediction of wild-type and mutant PHEX.


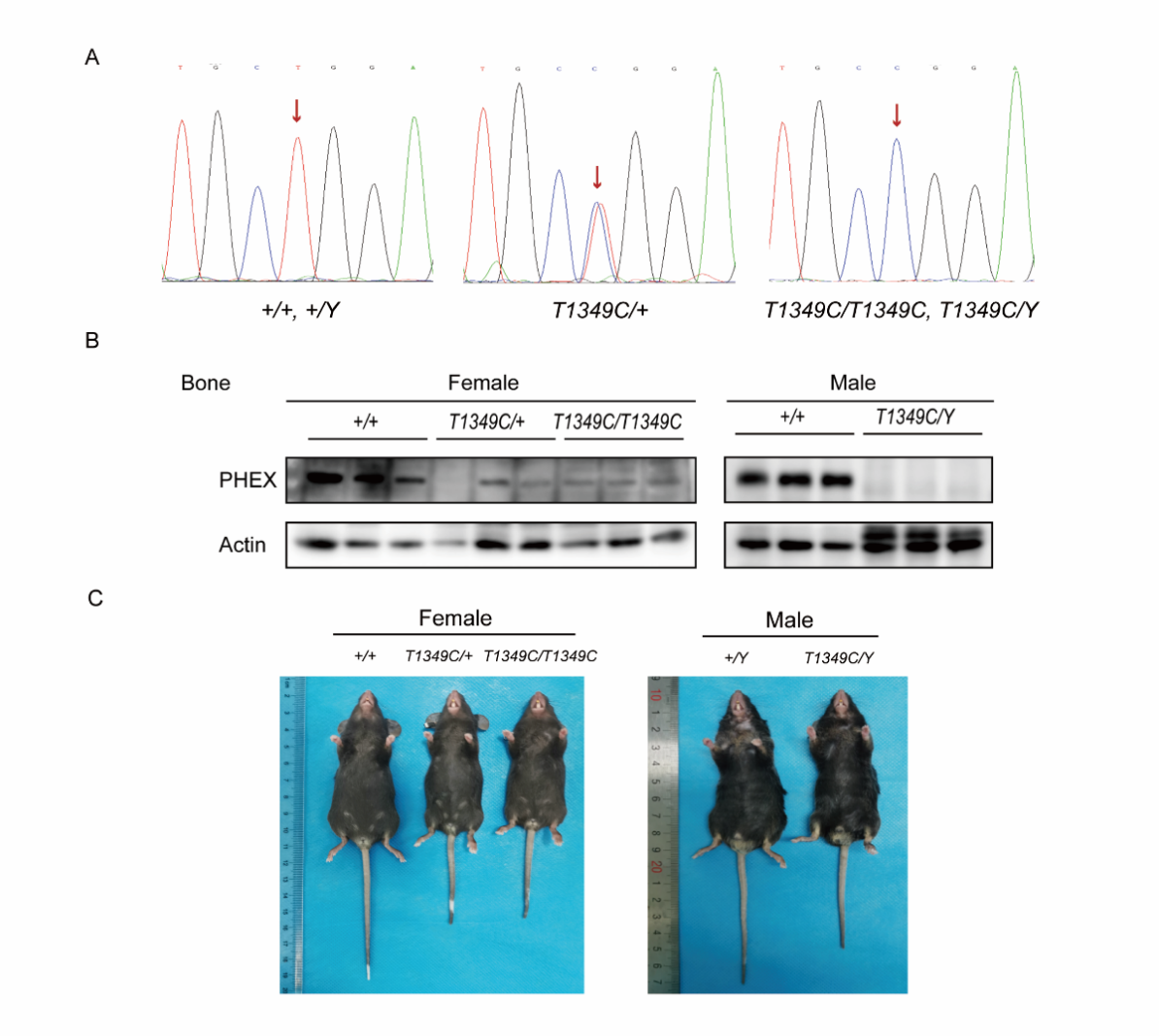


**Supplement Figure 3. *Phex*^T1349C^ mutation and its effect on *Phex* expression level in mice.** (A) Genomic sequence of the *Phex* gene spanning the mutation site (middle: heterozygous female; right: homozygous female and hemizygous male) and the corresponding site in the WT littermate (left). (B) Immunoblot analysis of protein from tibiae of mutant heterozygous (*Phex*^T1349C^/+) and homozygous (*Phex*^T1349C^/*Phex*^T1349C^) female mice, and hemizygous male (*Phex*^T1349C^/Y), and WT littermate using anti-PHEX antibody (top) and anti-β-actin antibody (bottom). (C) Images of *Phex*-T1349C mice compared to WT littermates of female (left image) and male (right image) mice at 12 weeks of age.


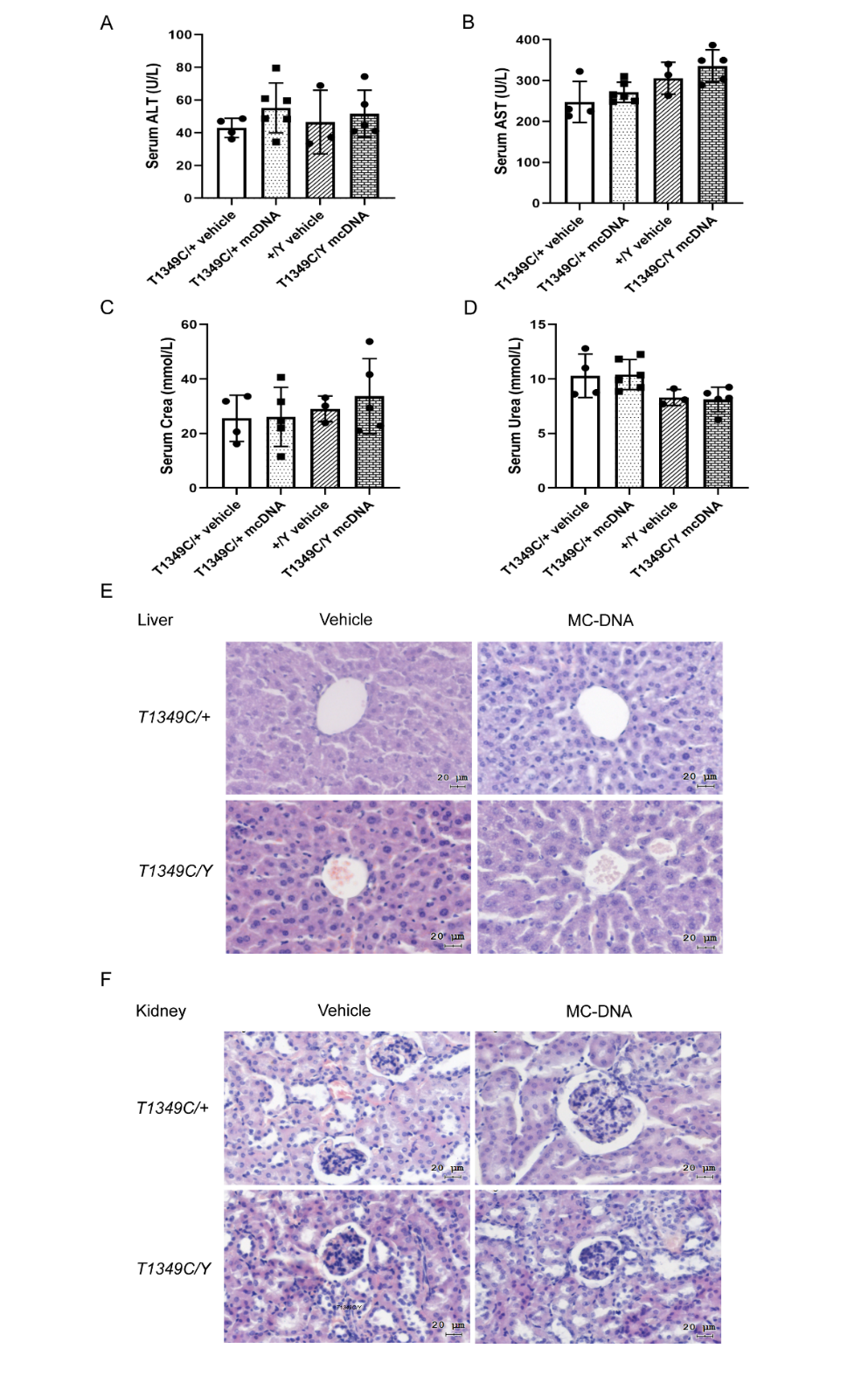


**Supplement Figure 4. Lack of hepatotoxicity and nephrotoxicity in *Phex*-T1349C mice treated with MC.CMV-FGF23 (180-251) vector.** Serum ALT (A) and AST (B) levels after injection with either vehicle or MC.CMV-FGF23 (180-251) vector. Serum Crea (C) and BUN (D) levels after injection with either vehicle or MC.CMV-FGF23 (180-251) vector. (E) Hematoxylin and eosin staining of liver tissues in vehicle and MC.CMV-FGF23 (180-251) group. (F) Hematoxylin and eosin staining of kidney tissues in vehicle and MC.CMV-FGF23 (180-251) group. Bars depict the mean±SEM (N=3-5 each group), p*＜0.05, p**＜0.001 by Student’s *t*-test for normally distributed continuous variables with homogeneous variance, or Mann Whitney test for non-normally distributed continuous variables with non-homogeneous variance.

**
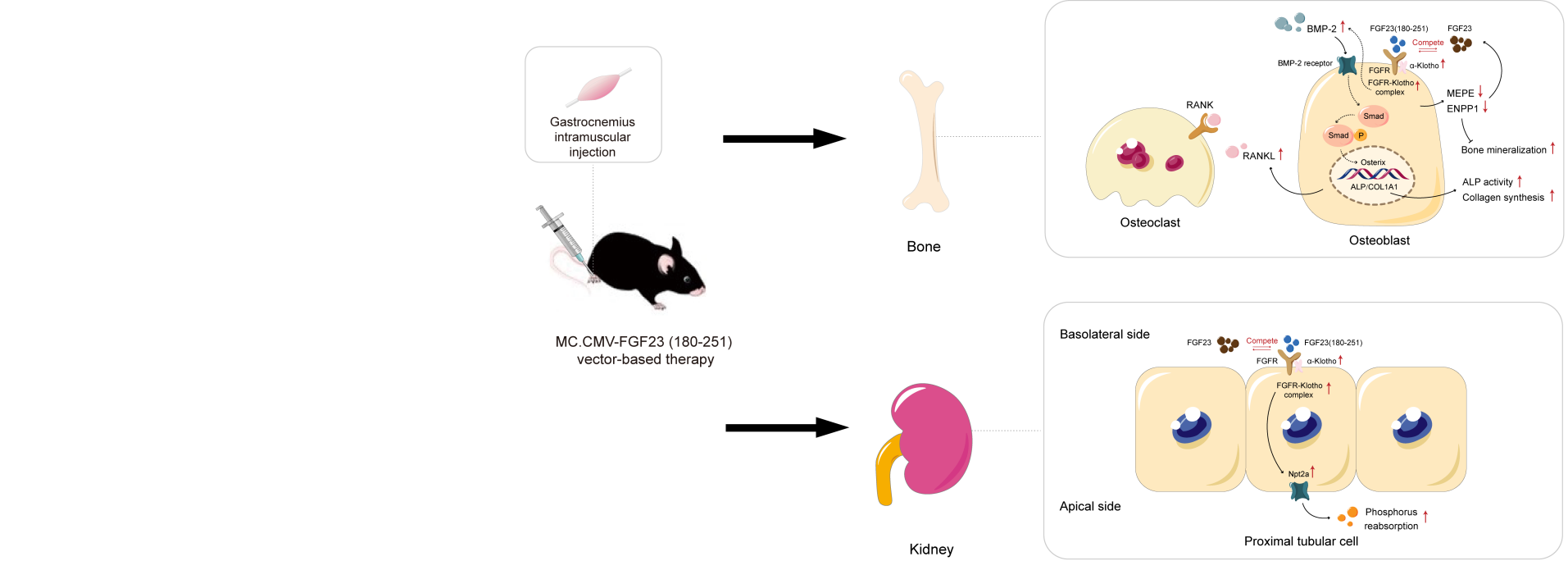
**

**Supplement Figure 5. A suggested scheme for MC.CMV-FGF23 (180-251) vector-based therapy in treated *Phex*-T1349C mice.**

**Movie S1. Video recording showing the phenotypic differences (small body size, short limbs/tail, decreased activity and waddling gait) between WT and *Phex*-T1349C male mice.** The bigger one is WT and the smaller one is T1349C/Y.

**Movie S2. Video recording showing the phenotypic differences (small body size, short limbs/tail, decreased activity and waddling gait) between WT and *Phex*-T1349C female mice.** The biggest one is WT, the middle one is T1349C/+ and the smallest one is T1349C/T1349C.
